# The sea cucumber genome provides insights into morphological evolution and visceral regeneration

**DOI:** 10.1371/journal.pbio.2003790

**Published:** 2017-10-12

**Authors:** Xiaojun Zhang, Lina Sun, Jianbo Yuan, Yamin Sun, Yi Gao, Libin Zhang, Shihao Li, Hui Dai, Jean-François Hamel, Chengzhang Liu, Yang Yu, Shilin Liu, Wenchao Lin, Kaimin Guo, Songjun Jin, Peng Xu, Kenneth B. Storey, Pin Huan, Tao Zhang, Yi Zhou, Jiquan Zhang, Chenggang Lin, Xiaoni Li, Lili Xing, Da Huo, Mingzhe Sun, Lei Wang, Annie Mercier, Fuhua Li, Hongsheng Yang, Jianhai Xiang

**Affiliations:** 1 Key Laboratory of Experimental Marine Biology & Marine Ecology and Environmental Sciences, Institute of Oceanology, Chinese Academy of Sciences, Qingdao, China; 2 Laboratory for Marine Biology and Biotechnology & Marine Ecology and Environmental Science, Qingdao National Laboratory for Marine Science and Technology, Qingdao, China; 3 Tianjin Biochip Corporation, Tianjin, China; 4 Society for the Exploration and Valuing of the Environment (SEVE), Portugal Cove-St. Philips, Newfoundland, Canada; 5 College of Ocean and Earth Sciences, Xiamen University, Xiamen, China; 6 Department of Biology, Carleton University, Ottawa, Ontario, Canada; 7 University of Chinese Academy of Sciences, Beijing, China; 8 College of Life Sciences, Nankai University, Tianjin, China; 9 Department of Ocean Sciences, Memorial University, St. John's, Newfoundland, Canada; The Wellcome Trust Sanger Institute, United Kingdom of Great Britain and Northern Ireland

## Abstract

Apart from sharing common ancestry with chordates, sea cucumbers exhibit a unique morphology and exceptional regenerative capacity. Here we present the complete genome sequence of an economically important sea cucumber, *A*. *japonicus*, generated using Illumina and PacBio platforms, to achieve an assembly of approximately 805 Mb (contig N50 of 190 Kb and scaffold N50 of 486 Kb), with 30,350 protein-coding genes and high continuity. We used this resource to explore key genetic mechanisms behind the unique biological characters of sea cucumbers. Phylogenetic and comparative genomic analyses revealed the presence of marker genes associated with notochord and gill slits, suggesting that these chordate features were present in ancestral echinoderms. The unique shape and weak mineralization of the sea cucumber adult body were also preliminarily explained by the contraction of biomineralization genes. Genome, transcriptome, and proteome analyses of organ regrowth after induced evisceration provided insight into the molecular underpinnings of visceral regeneration, including a specific tandem-duplicated prostatic secretory protein of 94 amino acids (PSP94)-like gene family and a significantly expanded fibrinogen-related protein (FREP) gene family. This high-quality genome resource will provide a useful framework for future research into biological processes and evolution in deuterostomes, including remarkable regenerative abilities that could have medical applications. Moreover, the multiomics data will be of prime value for commercial sea cucumber breeding programs.

## Author summary

Echinoderms, ubiquitous in the marine environment, are important from evolutionary, ecological, and socioeconomic perspectives. Together with chordates and hemichordates, they form the deuterostome clade, making them a crucial node in the study of chordate ancestry. Within echinoderms, class Holothuroidea is unique; its members (the sea cucumbers) display remarkable regenerative abilities and play key roles as sediment bioturbators and symbiotic hosts, and many are prized in the seafood and pharmaceutical industries. The sea cucumber genome therefore has the potential to significantly contribute to our understanding of important evolutionary and biological processes and help enhance aquaculture programs. Here we present a high-quality genome sequence for the economically important species *Apostichopus japonicus*. Through comparative analyses, we identified 763 echinoderm-specific gene families enriched in genes encoding membrane proteins, ion channels, and signal transduction proteins. Marker genes associated with the notochord and gill slits were also found, providing valuable insight into the origin of chordates. The reduced number and low expression levels of biomineralization genes reflect the skeletal degeneration seen in sea cucumbers. Importantly, 2 gene families appeared to be expanded in *A*. *japonicus* and may play crucial roles in its heightened regenerative potential. Together, findings from the sea cucumber genome provide important and novel insights into echinoderm and deuterostome biology.

## Introduction

Echinodermata, an ancient phylum of marine invertebrates, comprises 5 extant classes, including Echinoidea (sea urchins), Asteroidea (sea stars), Holothuroidea (sea cucumbers), Ophiuroidea (brittle stars), and Crinoidea (sea lilies). Together, the phyla Echinodermata, Hemichordata, and Chordata form the deuterostome clade, based on their closely shared developmental features. To date, 2 complete echinoderm genomes, that of the sea urchin *Strongylocentrotus purpuratus* and that of the sea star *Acanthaster planci*, have been successfully sequenced [1,2]. However, because sea cucumbers are unique among echinoderms, possessing many distinctive biological characteristics, their genome holds invaluable insight that can extend the scope and depth of molecular research in Echinodermata and Deuterostomia.

Sea cucumber adults exhibit an elongated shape that belies their pentaradial symmetry, combined with weak calcification in the form of microscopic ossicles that contrasts with the solid calcified test of sea urchins. Exploring these features can help in exploring the evolution of mineralization in echinoderms, which remains poorly understood.

Of even greater interest is the fact that sea cucumbers display a capacity to regrow body parts and internal organs [3], which is much greater than that of sea stars and sea urchins, making them prime regeneration models. The use of *A*. *japonicus* in this field is facilitated by its natural ability to discard its internal organs, rapidly regenerate them, and restore normal functions within a few weeks, through a process that involves cell migration, proliferation, differentiation, and organ/tissue reconstruction [3***–***5]. Finally, sea cucumbers, like many echinoderms, can be extremely long-lived and somewhat immune to senescence [6,7]. Therefore, knowledge of the complete genome of a sea cucumber will provide a unique framework for studies that seek to understand cell and tissue regeneration, treat organ failure, and alleviate the symptoms of aging.

Sea cucumbers are also widespread, occurring from the shore to the abyss, and can represent up to 80% of the whole biomass of benthic invertebrates in some areas. They are the target of important fisheries and represent the fastest-growing aquaculture sector worldwide [8]. However, overfishing and poor management of these valuable resources are a growing concern [8,9].

The sea cucumber *A*. *japonicus* is one of the most studied echinoderms; it is being cultivated commercially on a large scale in the western North Pacific Ocean and is one of the most valuable sea foods worldwide, due to its potent nutritional and medicinal properties [10***–***12]. In China alone, around 200,000 tons of sea cucumbers were produced in 2015, with an estimated value of about 4,000,000,000 United States dollars [13]. Improving genomic knowledge of this sea cucumber may therefore benefit the seafood industry and concurrently yield pharmaceutical and biomedical breakthroughs.

In early 2017, a draft genome of *A*. *japonicus* was published that represented only about 80.5% of the estimated genome size (0.82 Gb), with scaffolds N50 value of 10.5 Kb [14]. These data provided an important resource for sea cucumber genomics, but their incompleteness and fragmentation limit applications for research. Here we present a high-quality reference genome of *A*. *japonicus* investigated through a multiomics approach, providing valuable insights into the molecular and genomic basis of crucial evolutionary traits in sea cucumbers and deuterostomes. Knowledge of the complete genome of a holothuroid offers a particularly useful framework for studies that seek to understand the mechanisms of cell and tissue/organ regeneration.

## Results and discussion

### The sea cucumber reference genome

#### Genome assembly

**Fig 1:**
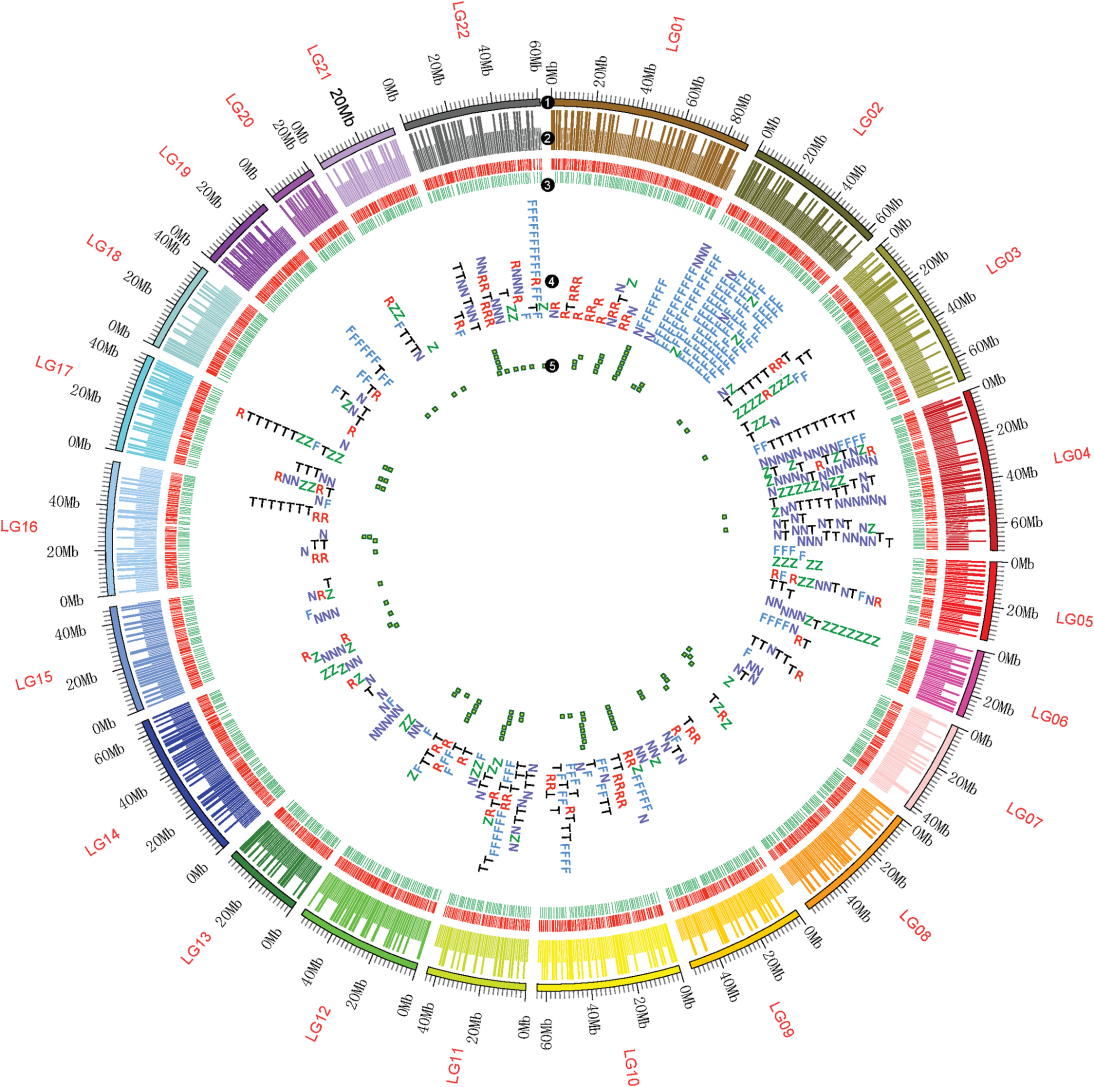
A schematic representation of the genomic characteristics of *A*. *japonicus*. Track 1: 22 linkage groups of the genome. Track 2: anchored scaffolds to each linkage group. Long bars represent scaffolds with a length > 500 Kb; short bars represent scaffolds with a length ≤ 500 Kb. Track 3: protein-coding genes located on scaffolds. Red stands for genes on the forward strand, and green stands for genes on the reverse strand. Track 4: distributions of 5 significantly expanded gene families in the genome. The 5 gene families are fibrinogen-related proteins (FREPs) (F), retrovirus-related Pol polyprotein from transposon (R), nucleotide-binding oligomerization domain-like receptors (NLR) family caspase recruitment domain (CARD) domain-containing protein (N), tyrosine-protein kinase receptor (T), and zinc finger CysCysHisCys (CCHC) domain-containing protein (Z). These expanded gene families show a clustered distribution in the genome. FREPs mainly clustered at LG02, and NLR chiefly accumulated at LG04. Track 5: distribution of microRNA (miRNA) in the genome. Most miRNAs are distributed in clusters across the genome (LG01, LG10, LG11, LG12, and LG22). The data underlying Fig 1 can be found in S3 Data.

The genome of *A*. *japonicus* was sequenced using a combination of Illumina shot-gun and PacBio Single Molecule Real-Time (SMRT) sequencing. A total of 260 Gb (approximately 294.97× coverage) of Illumina clean data and approximately 64 Gb (approximately 72.66× coverage) of long subreads from SMRT sequencing were obtained for the genome assembly (S1 and S2 Tables). A high level of heterozygosity is one of the main challenges of genome assembly in marine invertebrates. Initially, the whole-genome shotgun strategy resulted in an assembled genome that was highly fragmented. With the benefit of PacBio sequencing, genome assembly was performed with 4 different newly developed approaches (S3 Table), and we selected the most performant software (Fast Alignment and Consensus for Assembly [FALCON]) for the contig construction, with Illumina mate-pair data integrated for the scaffold extension. Finally, we obtained a reference genome with the assembly of 804.99 Mb, which covered approximately 91.47% of the genome size estimated from flow cytometry (880.20 ± 58.68 Mb) [15]. The assembled contigs displayed high continuity with an N50 length of 190 Kb, which is longer than that of other marine invertebrates whose genomes are available (S4 Table), such as *S*. *purpuratus* (18 Kb) and *A*. *planci* (55 Kb). A total of 3,821 scaffolds were assembled with an N50 length of 486 Kb, and approximately 90% of the total assembled genome consisted of the 1,779 longest scaffolds (Table 1 and S5 Table). A high-density genetic linkage map with 6,350 high-quality markers was used to position and orient 1,313 scaffolds (573.32 Mb in length, 71.2% of genome) into 22 linkage groups (Fig 1).

**Table 1:** Summary of *A*. *japonicus* genome assembly.

**Genome assembly statistics**		
Total length	804,993,085 bp
Number of scaffolds	3,281
Largest contig length	1,074,537 bp
Largest scaffold length	2,494,933 bp
N50 length (contigs)	190,269 bp
N50 length (scaffolds)	486,650 bp
N90 length (scaffolds)	121,462 bp
Number of scaffolds (>N90 length)	1,779
**Genome characteristics**		
GC content	36.75%
Predicted heterozygosity	1.59%
Content of transposable elements	27.20%
Predicted protein-coding gene number	30,350
Predicted noncoding RNA gene number	1,754
Quantity of scaffolds anchored on linkage groups	1,313
Length of scaffolds anchored on linkage groups	573.32 Mb

Abbreviations: GC content, guanine-cytosine content.

A comparison of the assembly results with different coverage (30× to 70×) of the PacBio data showed that 70-fold coverage was sufficient to cover the full-length genome (S1 Fig, S3 Table). Over 94% of reads from the Illumina short-insert library could be successfully mapped to the genome; moreover, over 97% of transcriptome unigenes were mapped to the assembly, and over 93% of unigenes were covered by a single scaffold at half length (50% in 1 scaffold) (S6 Table), reflecting the high integrity and accuracy of the assembly. Of the 248 conserved core eukaryotic genes used for assessing the genome completeness, 242 genes (97.6%) were covered by the genome (S7 Table). These data show that the assembled *A*. *japonicus* genome is complete for protein-coding sequences.

#### Genome structure characteristics

The genome of *A*. *japonicus* showed approximately 1.59% heterozygosity based on k-mer analysis of short-insert library reads (S3 Fig), which was comparable to other highly polymorphic species, such as *Hydra magnipapillata* (0.70%), *Ciona intestinalis* (1.20%), and *Crassostrea gigas* (1.3% for wild type, 0.73% for inbred type) [16–18]. Moreover, we identified a total of 210.87 Mb (26.20%) of the assembled genome as transposable elements. Of these, 7.10% could be annotated with known repeat families, and 18.93% were specific unknown repeats, which were similar to results obtained from *S*. *purpuratus* and *A*. *planci* (S8 Table). However, *A*. *japonicus* and *A*. *planci* have fewer DNA transposons (3.20% and 2.98%, respectively) than *S*. *purpuratus* (8.36%).

Various noncoding small RNAs were found in the sea cucumber genome, including 1,127 tRNAs, 223 small nuclear RNAs (snRNAs), 149 small nucleolar RNAs (snoRNAs), 137 microRNAs (miRNAs), and 75 rRNAs (S1 Data). The 137 miRNAs can be clustered into 76 families, including 39 conserved and 37 novel families, among which miR-92 (with 12 copies) was the most abundant. Moreover, most of the miRNAs are distributed across the genome in clusters (12 clusters), in which 2 or more miRNAs are located in physically adjacent regions, indicating possible cotranscription and functional cooperation (Fig 1).

A total of 30,350 protein-coding gene models were constructed, and among them, 28,144 (92.73%) were supported by the transcriptome data. Compared to *S*. *purpuratus* and *A*. *planci*, *A*. *japonicus* has a similar average exon size (193.43 bp) and exon number per gene (6.1), but it has a shorter intron size (1,319 bp on average) than *S*. *purpuratus* (S5 Fig and S9 Table).

### Genomic analyses shed light on morphological evolution in deuterostomes

#### Phylogenetic analyses

**Fig 2:**
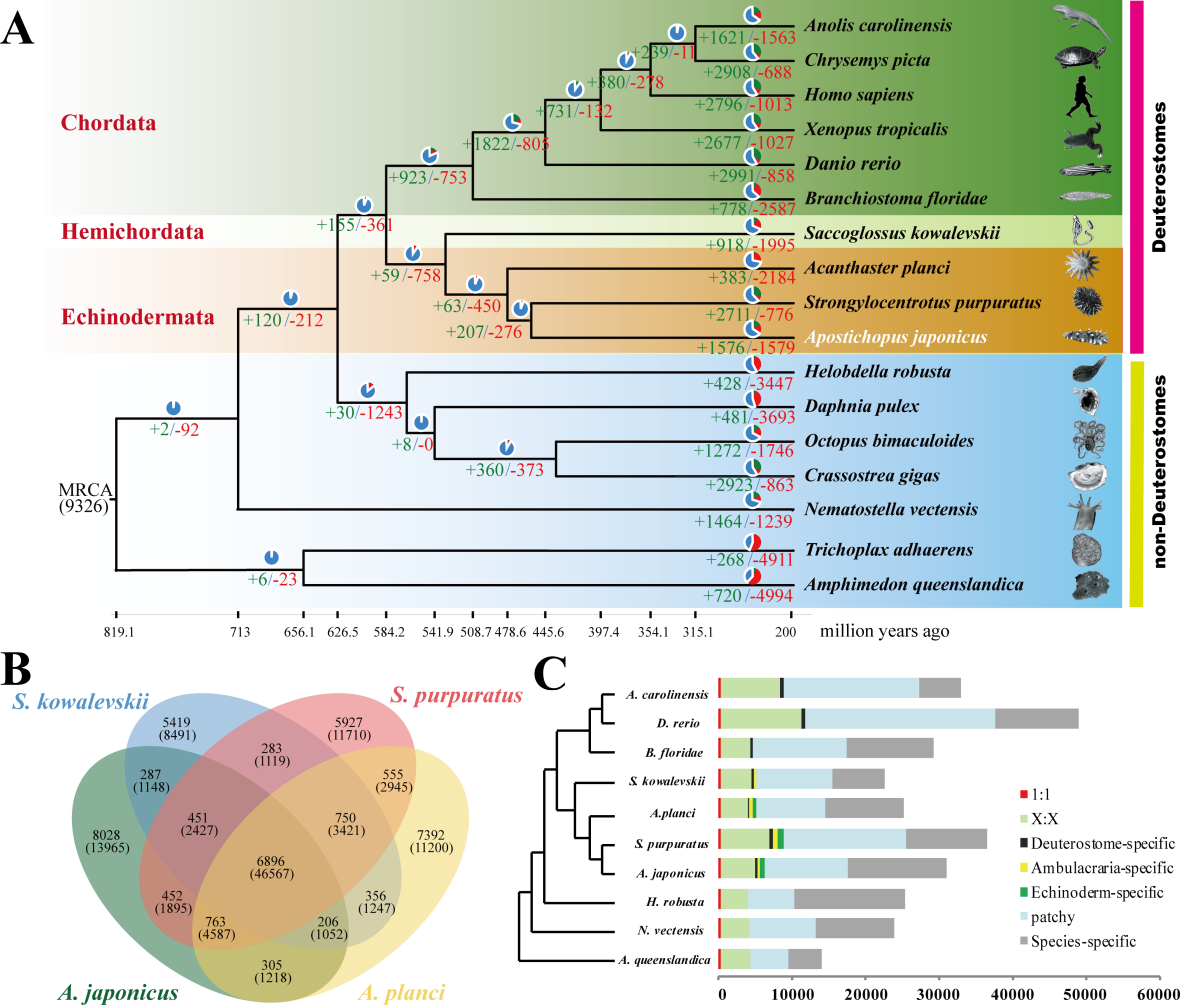
Comparative genomic analysis between *A*. *japonicus* and other metazoans. (A) Phylogenetic placement of *A*. *japonicus* within the metazoan tree. The numbers on the branches indicate the number of gene gains (+) and the number of gene losses (−), which are also displayed as bar plots: gene gain (in green), gene loss (in red), and the remaining gene families (in blue). The divergence times were estimated and displayed below the phylogenetic tree. Image credits: Robert Michniewicz; Kobie Mercury-Clarke; Vector Open Stock, Patrick Narbonne, David E. Simpson, John B. Gurdon; Lars Simonsen; Freshwater and Marine Image Bank; Encyclopædia Britannica; public domain; Jerry Kirkhart; authors' own; Johny Ha; Virginia Gewin; public domain; public domain; Cnidaria; Michael Eitel, Hans-Jürgen Osigus, Rob DeSalle, Bernd Schierwater; Maja Adamska. (B) The shared and unique gene families in 4 species of Ambulacraria are shown in the Venn diagram. There are 763 gene families shared by 3 echinoderms. (C) Comparison of the gene repertoire of 10 metazoan genomes. Here, "1:1" indicates single-copy genes; "X:X" indicates orthologous genes present in multiple copies in all the 9 species, where X means 1 or more orthologs per species; and "patchy" indicates the existence of other orthologs that are present in at least 1 genome. *A*. *japonicus* and *S*. *purpuratus* show a similar distribution of gene repertoire. The data underlying Fig 2 can be found in S3 Data.

To understand the phylogenetic location of sea cucumbers, we used a maximum likelihood method for genome-wide phylogenetic analysis based on single-copy genes from 17 genomes (Fig 2A). The results support the view that echinoderms and hemichordates (*Saccoglossus kowalevskii*) are sister groups and share a common Ambulacraria ancestor, which is the basal taxon of deuterostomes. Based on phylogeny and fossil records, we dated the divergence time of hemichordates and echinoderms to about 533 million years ago (Mya), which roughly coincides with the lower Cambrian evolutionary explosion. Moreover, phylogenomic analysis of the 5 echinoderm classes supported that sea cucumber (holothuroid) was a sister group to sea urchin (echinoid) and that the sea lily (crinoid) was the basal taxon of echinoderms (S6 Fig) [19]. The 3 echinoderms, *S*. *purpuratus*, *A*. *planci*, and *A*. *japonicus*, were estimated to have diverged approximately 479 Mya, suggesting a long evolutionary history for the 5 echinoderm classes after their divergence.

We performed comparative genomic analyses among 17 metazoan species and detected 49,351 families of homologous genes. Owing to gene loss and duplication, species-specific genes occupied a large part of genes, and few deuterostome-specific and Ambulacraria-specific genes were identified, while some Echinoderm-specific genes were cumulated (Fig 2C). There were 763 echinoderm-specific gene families shared by 3 echinoderms, which were enriched in the genes encoding membrane proteins, ion channels, and proteins involved in signal transduction (S10 Table). A total of 452 gene families were shared by *A*. *japonicus* and *S*. *purpuratus* (Fig 2B), many of them enriched in metabolism pathways of glycerophospholipid (*P* = 2.2E-04), arachidonic acid (*P* = 9.6E-04), and steroid hormone (*P* = 5.2E-03). Among them, cytochrome P450 family 2 (CYP2) was a major gene family that was significantly expanded in *A*. *japonicus* and *S*. *purpuratus* (S7 Fig). However, 13,103 genes in *A*. *japonicus* could not be grouped with any genes from the other 16 species; these genes were referred to as orphan genes. In total, 1,576 gene families were expanded in *A*. *japonicus* (S8 Fig), whereas 1,579 gene families were contracted (*P* < 0.01) (Fig 2A). The gene families expanded in the genome of *A*. *japonicus* were chiefly enriched in categories of signal recognition and immunity (S11–S15 Tables and S8 Fig), and gene repertoires showed some differences in the contents of multiple-copy genes and Echinoderm-specific genes (Fig 2C).

#### Notochord and gill slits

**Fig 3:**
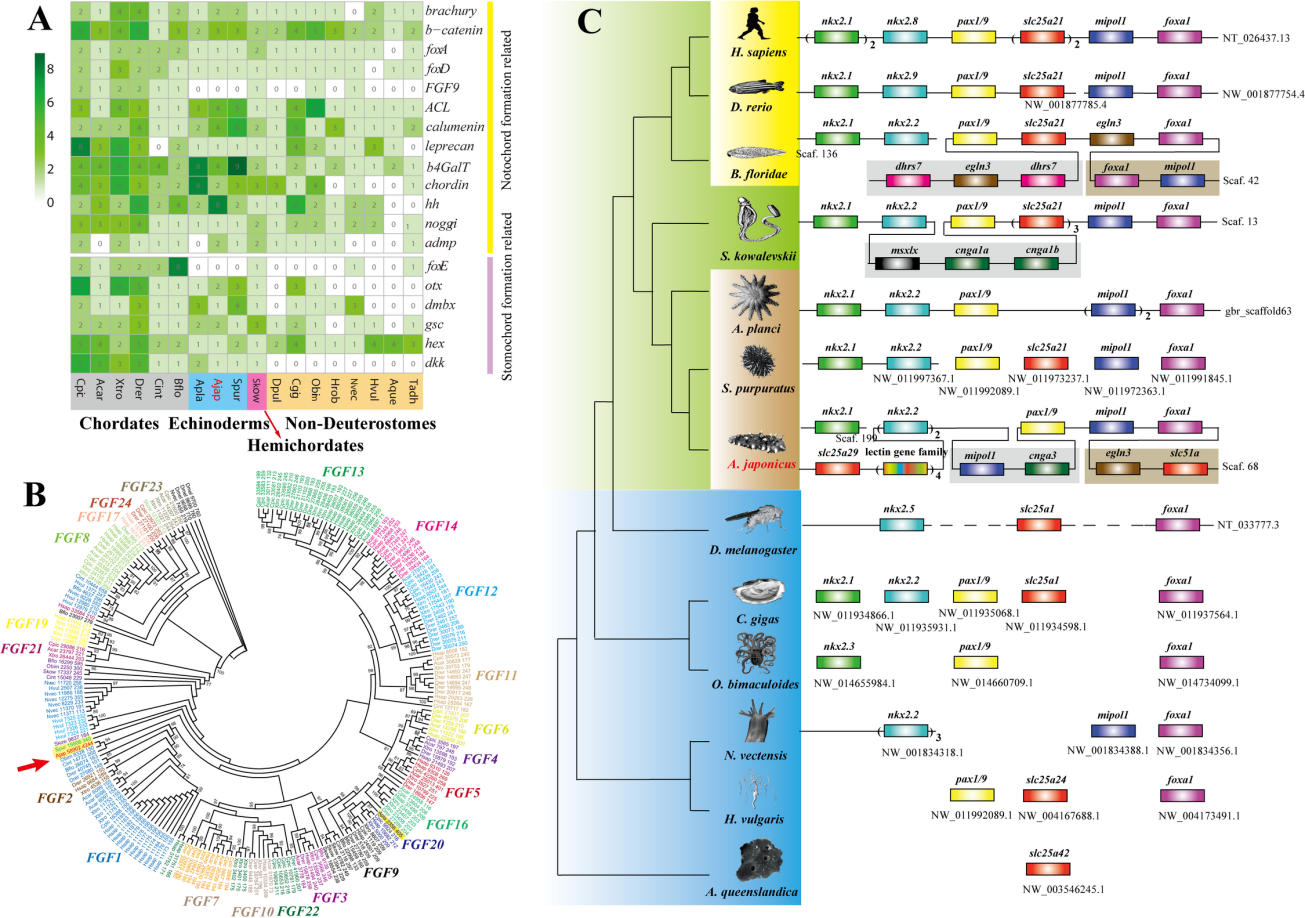
Gene families and gene clusters related to notochord and pharyngeal gill slit formation. (A) Distribution pattern of genes related to notochord and stomochord formation in metazoans. These genes are all present in deuterostomes, but some of them are present in nondeuterostomes. (B) Phylogenetic analysis of the fibroblast growth factor (FGF) gene family in metazoans. Different genes of the FGF gene family are distinguished with different colors. The FGF gene family is important for transcriptional activation of *bruchury* in notochord formation, but only 1 such gene exists in the genome of *A*. *japonicus* (red arrow) and the other 2 echinoderms (yellow background), *S*. *purpuratus* (Spur_15908_340) and *A*. *planci* (Apla_2356_405). The data underlying Fig 3A and 3B can be found in S3 Data. (C) A gene cluster related to the pharyngeal gill slit. The genes connected on a line indicate that they are clustered in order in the genome. This gene cluster is conserved among deuterostomes, whereas it is incomplete and shows poor synteny within nondeuterostomes. Image credits: Robert Michniewicz; Kobie Mercury-Clarke; Patrick Narbonne, David E. Simpson, John B. Gurdon; Vector Open Stock; Lars Simonsen; Freshwater and Marine Image Bank; Encyclopædia Britannica; public domain; Jerry Kirkhart; authors' own; Martin Cooper; public domain; public domain; Cnidaria; Freshwater and Marine Image Bank; Maja Adamska; Johny Ha; Virginia Gewin; Michael Eitel, Hans-Jürgen Osigus, Rob DeSalle, Bernd Schierwater.

Unlike hemichordates and chordates, adult echinoderms lack the ancestral deuterostome characters of gill slits and notochord. However, fossil evidence supports vetulocystids as the ancestral echinoderms, since they possess gill slits like hemichordates [20]. Some fossil evidences, such as stylophorans and *Jaekelocarpus*, share calcite skeletons with extant echinoderms and also share gill slits with chordates, suggesting a close relationship between echinoderms and chordates [21–23]. Furthermore, we found that most marker genes associated with notochord formation and gill slit development (e.g., *brachyury*, *β-catenin*, *foxA*, and *otx*) were commonly present in echinoderms, whereas some of them were absent in the nondeuterostome species (Fig 3A and 3C). Two conserved gene clusters related to pharyngeal gill slit development (*pax1/9*~*foxA* and *six6*~*six4*) showed strong synteny within deuterostomes [24], whereas they were incomplete and showed poor synteny within nondeuterostomes (Fig 3C and S10 Fig).

The transcription factor Brachyury is a key regulator of notochord formation in chordates, and its transcriptional activation is regulated by fibroblast growth factor (FGF) signaling pathway genes [25]. However, FGF gene families contracted significantly in echinoderms, such that only 1 FGF gene (*fgf8*) exists in *A*. *japonicus*, *S*. *purpuratus*, and *A*. *planci* (Fig 3B). Transcriptome data are consistent with this opinion, showing only 1 FGF gene expressed in *A*. *japonicus*. Similar to other chordates, *brachury* and *FGF* start to express at the gastrula stage (S11 Fig). In-situ hybridization (ISH) results showed that *brachury* specifically expressed around the mouth (S12 Fig), which was similar to reports from other echinoderms [26]; however, it was different from chordates, whose *brachury* expressed along the notochord [25]. Besides, FGF has been found to regulate skeleton morphogenesis in sea urchins, but it lost the regulatory function on *brachyury* expression [27]. The stomochord was considered as the supporting organ of hemichordates, even though no molecular evidence of homology has been detected between stomochord and notochord [28,29]. The marker gene of stomochord formation (*foxE*) is specifically expressed in the pharyngeal region of *S*. *kowalevskii* [30]. However, echinoderms have not evolved any tissues equivalent to the stomochord, and *foxE* was absent in all sequenced echinoderms (including sea star and brittle star) (Fig 3A and S16 Table). As for the gene cluster related to pharyngeal gill slit development (pax1/9~foxA), *Nkx2*.*1*, *FoxA*, and *Pax1/9* showed similar expression patterns that were highly expressed at the stages of gastrula and doliolaria, but other genes displayed variable expression patterns, and *Slc25A21* even displayed a reverse expression pattern (S13 Fig). In summary, the genomic characteristics related to notochord and gill slits in *A*. *japonicus* bear similarities and some differences to chordates, suggesting that these features might have been present in ancestral echinoderms but subsequently lost.

#### Homeobox genes

**Fig 4:**
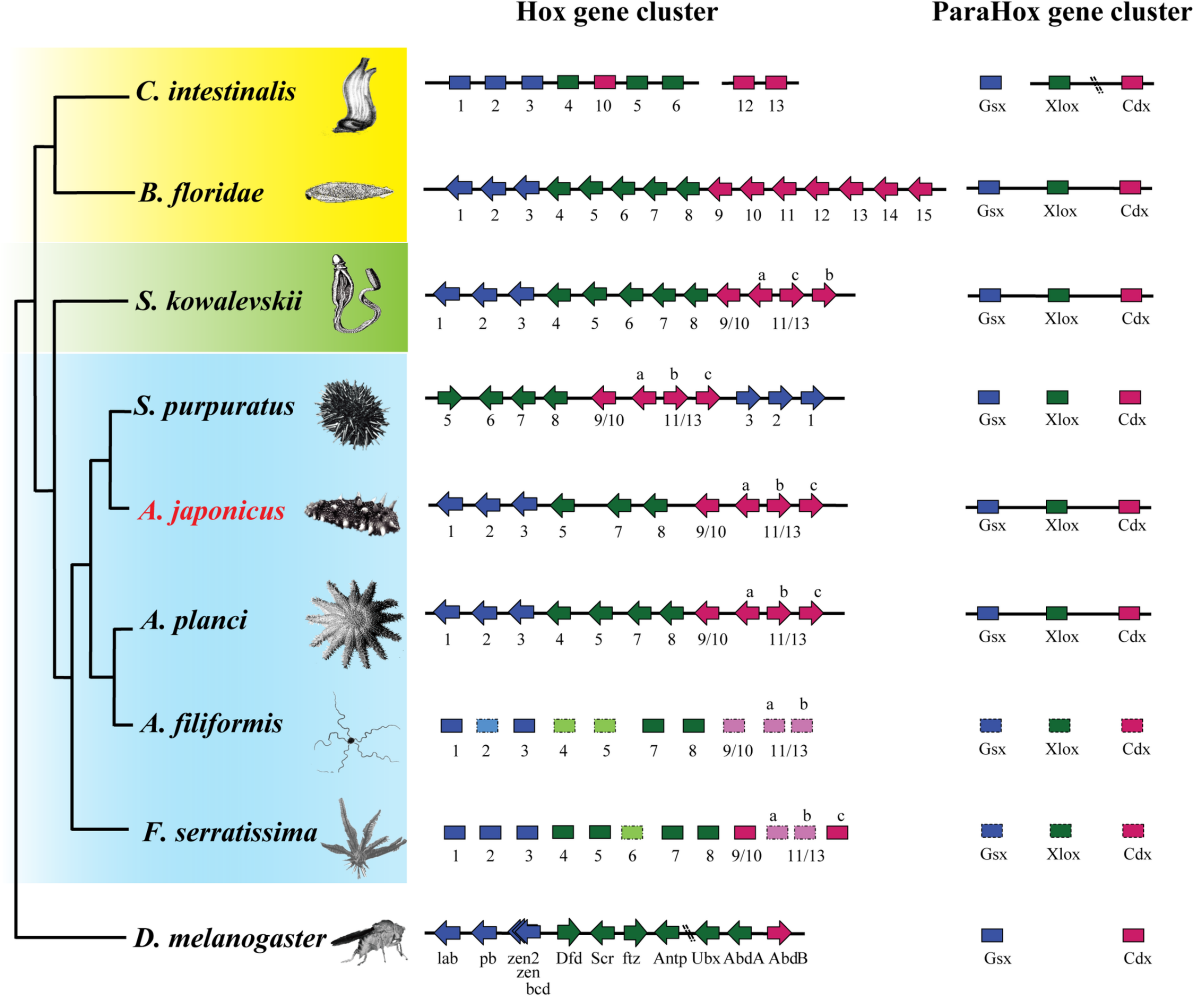
The conserved homeobox gene clusters in the genome of *A*. *japonicus*. *A*. *planci*, *Amphiura filiformis*, and *Florometra serratissima* belong to Echinodermata class Asteroidea, Ophiuroidea, and Crinoidea, respectively. The general phylogenetic tree shows the Hox and ParaHox cluster order and genes. Hox4 and Hox6 are not found in the genome of *A*. *japonicus*. Different colors indicate different Hox gene groups, including anterior (blue), central (green), and posterior (red). The arrow indicates the direction of transcription. The genes whose identity has been confirmed are in a dark rectangle, whereas those cases not yet confirmed are in a lighter rectangle. The Hox cluster images of *Branchiostoma floridae*, *S*. *purpuratus*, *A*. *filiformis*, and *F*. *serratissima* were modified from Byrne et al. 2016 [34]. Image credits: "Introduction to zoology; a guide to the study of animals, for the use of secondary schools" (1900) Macmillan; Freshwater and Marine Image Bank; Encyclopædia Britannica; Jerry Kirkhart; authors' own; public domain; Freshwater and Marine Image Bank; National Oceanic and Atmospheric Administration; Martin Cooper.

The Hox gene cluster is widely known for its role in patterning the anterior-posterior axis of animal embryos. The ParaHox gene cluster, including *Gsx*, *Xlox*, and *cdx*, is the evolutionary sister of the Hox gene cluster. In the *A*. *japonicus* genome, we found a single, substantially intact, and well-ordered Hox gene cluster containing 10 Hox genes, as well as a conserved ParaHox gene cluster (Fig 4) and large numbers of other homeobox genes (S17 Table). The Hox gene cluster, *Hox1* to *Hox11/13c*, spanned approximately 940 Kb, which is similar to reports from acorn worms (*S*. *kowalevskii* and *Ptychodera flava*, approximately 500 Kb) [31], sea urchin (*S*. *purpuratus*, 588 Kb) [32], and sea star (*A*. *planci*, 1,195Kb) [33]. However, the gene order of Hox clusters in *A*. *japonicus* was similar to that of *A*. *planci* and different from that of *S*. *purpuratus*, whose anterior class genes (*Hox1*, *Hox2*, and *Hox3*) were inverted and translocated to the end of the cluster because of genomic rearrangement (Fig 4) [32]. Thus, the present results are consistent with the opinion that translocation and inversion (TAI) of the Hox cluster was not a feature of the Echinozoa (sea urchins and sea cucumbers), and the pentameral body plan was not linked to Hox gene arrangements in echinoderms [34]. *Hox4* and *Hox6* were not found in the *A*. *japonicus* genome. Generally, *Hox4* is conserved in Asteroidea, and its expression is characterized in the dwarf cushion star *Parvulastra exigua* [35]. However, *Hox4* is lost in *S*. *purpuratus* [32] whose *Hox6* was highly homologous to *A*. *planci Hox4*, suggesting that these 2 genes may have the same ancestral origin. A homeodomain of *Hox2* was found between *Hox1* and *Hox3* in the *A*. *japonicus* Hox cluster, but its gene structure was not intact. It could not be found in the available *A*. *japonicus* transcriptomes. The annotated *Hox2* sequence could not be amplified by PCR from embryonic and larval cDNA of *A*. *japonicus*. As there are no reports of their function in echinoderms, we speculate that the absence of these Hox genes may be involved in the unique body shape of the sea cucumber and other echinoderms.

#### Skeleton degeneration

**Fig 5:**
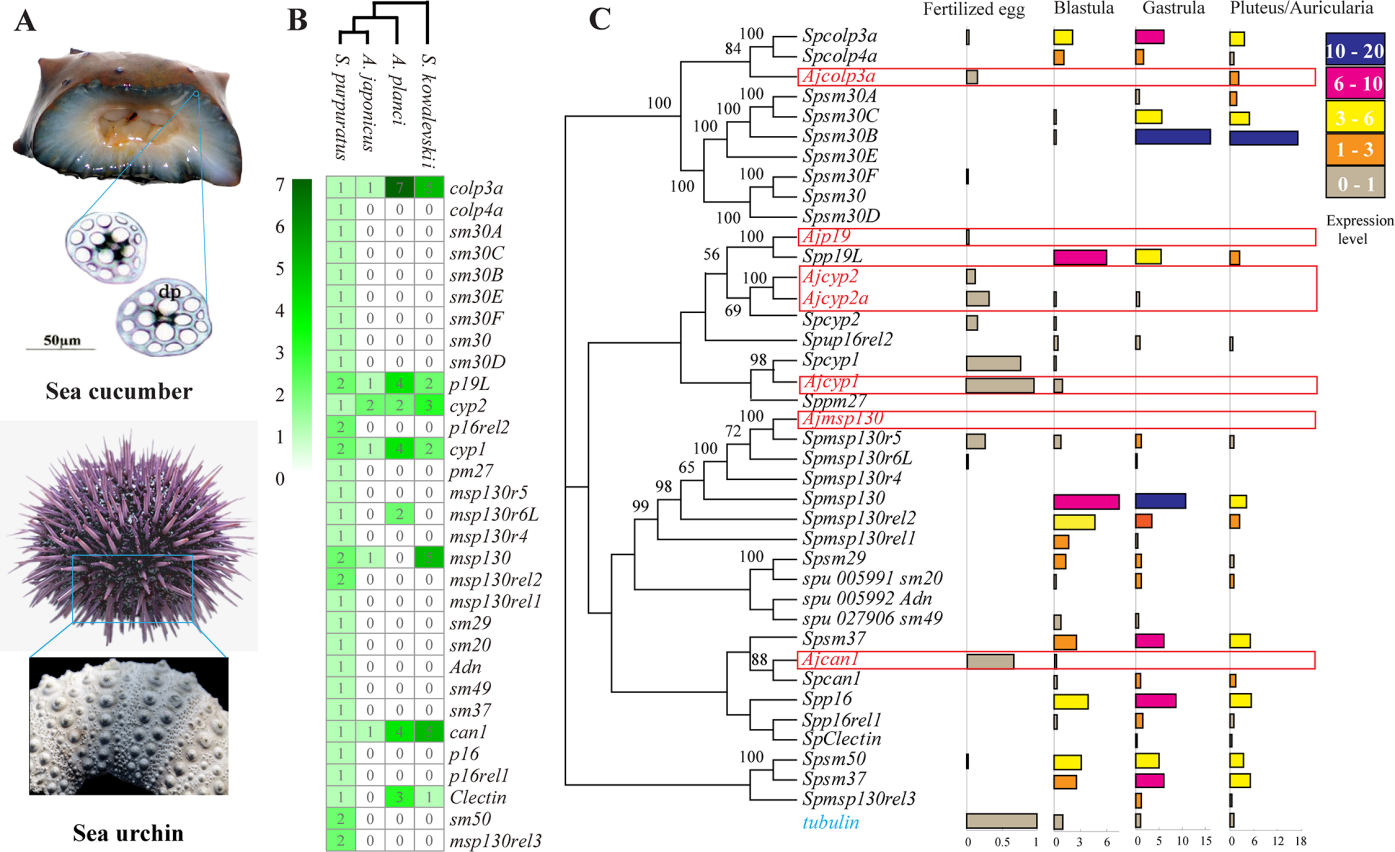
Gene families related to skeletogenesis and biomineralization in the genome of *A*. *japonicus*. (A) Comparison of the skeleton from *A*. *japonicus* and *S*. *purpuratus*. (B) The comparison of the copy numbers of biomineralization-related genes in 3 echinoderms, *A*. *japonicus*, *S*. *purpuratus*, and *A*. *planci*, and the hemichordate *S*. *kowalevskii*. Biomineralization-related genes are apparently contracted in *A*. *japonicus*. (C) The expression levels of biomineralization-related genes at early developmental stages of *A*. *japonicus* (red) and *S*. *purpuratus* (black). The expression level of these genes at different development stages is calculated relative to a housekeeping gene (*tubulin*). Compared with sea urchin, there is a significant gene loss in the *A*. *japonicus* genome, and the expression levels for those genes at different development stages are relatively low. The data underlying Fig 5B and 5C can be found in S3 Data.

Unlike other echinoderms with a well-developed calcareous endoskeleton, sea cucumbers have a soft body wall, and microscopic skeletal ossicles are imbedded into a thick layer of the dermal connective tissue (Fig 5A). In spite of its markedly reduced skeleton, the sea cucumber *A*. *japonicus* shares many components of the skeletogenic regulatory system with heavily calcified sea urchins and other echinoderms [36–38]. It has relatively intact skeletogenic signal pathways, key transcription factors, and important skeletogenesis-related genes (e.g., *otx*, *soxB*, *ets1*, *alx1*, *dlx*, *runx2*, *snail*, and *twist*) (S18 Table). However, the number of downstream biomineralization genes is significantly different between the sea urchin and the sea cucumber. Compared with 31 biomineralization genes reported in *S*. *purpuratus* [39], only 7 were found in *A*. *japonicus* (S18 Table). Further analysis of these genes in the sea star (*A*. *planci*) and acorn worm (*S*. *kowalevskii*) found that *colp3a*, *p19*, *cyp1*, *cyp2*, *msp130*, and *can1* are the common biomineralization genes in the 4 Ambulacraria animals, but these genes are fewer in *A*. *japonicus* than in the other 3 species, especially *S*. *purpuratus* (Fig 5B). Therefore, we speculated that biomineralization genes were contracted in sea cucumbers and significantly expanded in sea urchins (Fig 5B). The transcriptome data revealed that most biomineralization genes were highly expressed in *S*. *purpuratus* from the blastula (embryo) through the pluteus (larva) stages, whereas the 7 corresponding biomineralization genes in *A*. *japonicus* showed relatively lower expression levels in these developmental stages (Fig 5C). Biomineralization is a key process of skeleton formation, and mineralization gene expression directly determines the final scale of the skeleton. Most of these biomineralization genes (*Sm30* and *Sm50*) encode spicule matrix proteins that mediate the deposition of the initial calcite crystal granules [39,40]; the others encode collagens (*colp3a*), the mesenchyme-specific cell surface protein (*Msp130*), and the primary mesenchyme cell (PMC) specific carbonic anhydrase (*can1*) [41]. It appears that *A*. *japonicus* lacks the spicule matrix genes (such as the *Sm30* family). Therefore, the contraction of biomineralization genes likely underlies both the evolutionary and developmental degeneration of the sea cucumber’s skeleton.

#### Nervous system-related genes

Sea cucumbers have no true brain or classic sensory organs, but they have a primary central nervous system specifically, a radial nerve cord and various nerve endings scattered through the skin giving them a response to touch and sensitivity to light. The genome data supported these characteristics. Like sea urchins and sea stars, *A*. *japonicus* has a large number of genes related to the development of the nervous systems in its genome, whether in adult or larva [42]. However, some crucial genes related to central nervous system development were absent in *A*. *japonicus*, *S*. *purpuratus*, and *A*. *planci*, including *hunchback*, *vax*, *sax7*, *pax2*, *gfap*, *krox20*, *L-fng*, *Gli*, and *pax3/7* (S2 Data), suggesting that all echinoderms have a similar genomic comparison and an absence of central nervous system genes. One difference is that *A*. *japonicus* has some significantly expanded gene families mainly enriched in signal recognition and nervous system, such as tyrosine-protein kinase receptor and netrin receptor UNC5B (S11–S15 Tables). One of the most expanded gene families was the netrin receptor (234 genes), which expanded many folds compared to related species. The netrin receptor is the key regulator in neuronal cell growth and migration [43]. Therefore, the expansion of these gene families might be a compensatory adaptation of the response to the lack of a central nervous system in *A*. *japonicus*.

### The underpinnings of exceptional regenerative capacities in sea cucumbers

#### A novel regeneration-related gene cluster

**Fig 6:**
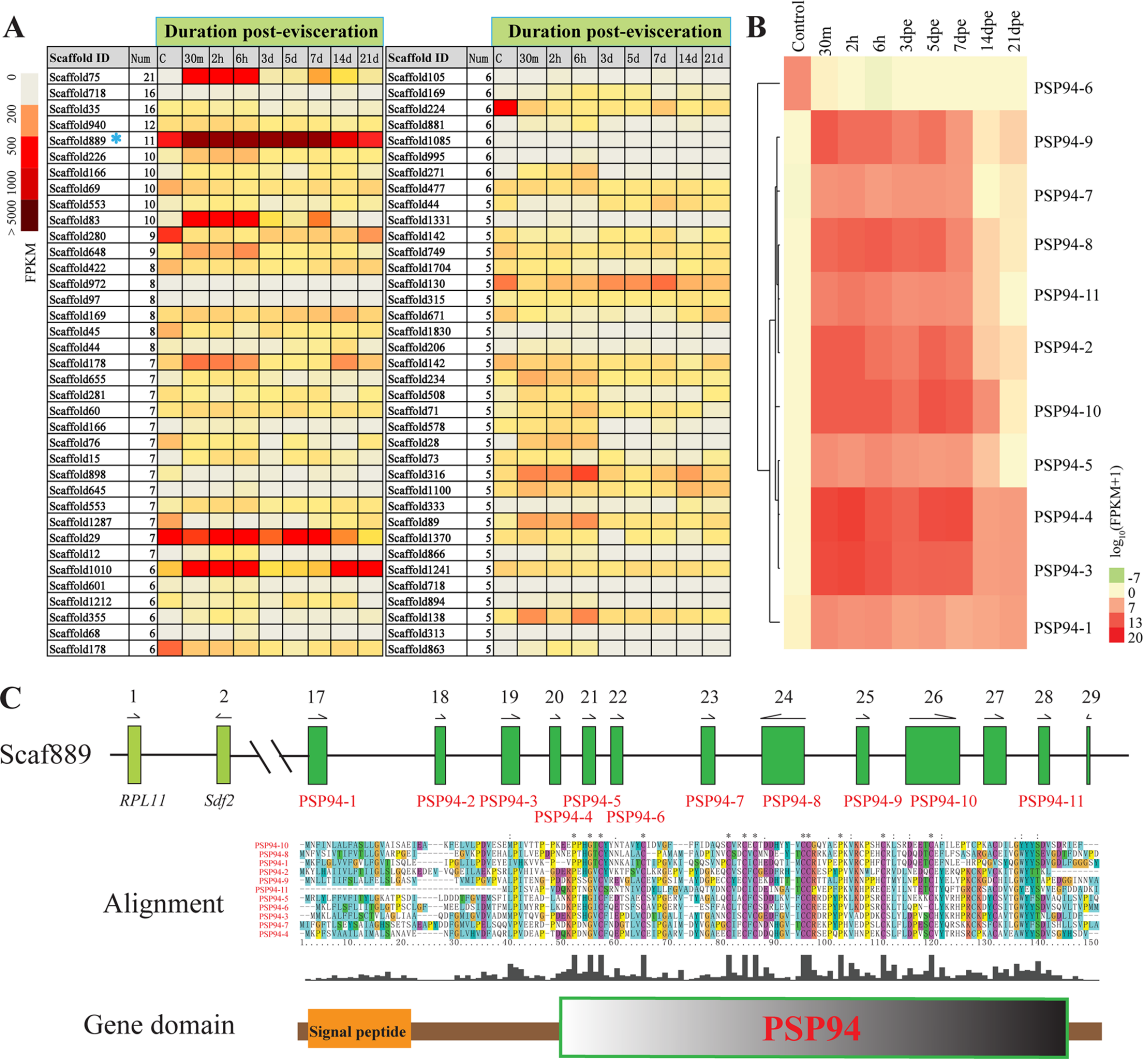
Prostatic secretory protein of 94 amino acids (PSP94)-like gene family in the genome of *A*. *japonicus*. (A) A heatmap showing the expression profile of tandem-duplicated gene clusters during the intestinal regeneration of *A*. *japonicus*. It depicts the cluster ID, the repeat number, and the expression profile. The color represents the relative expression level (fragments per kilobase of transcript per million mapped reads [FPKM] value) of the gene clusters during the intestinal regeneration. The Scaffold889 with 11 tandem-duplicated genes (blue asterisk) shows significant up-regulation post evisceration. (B) The detailed expression of 11 PSP94-like genes located on Scaffold889 at different time points post evisceration. The scale covers log expression values. Genes are clustered by Euclidean distance of the log_10_(FPKM+1) value and grouped with average-linkage clustering. (C) The distribution and alignment of the PSP94-like gene family. Eleven PSP94-like genes are tandemly arranged on Scaffold889. Arrows denote the transcriptional orientation. The amino acid sequence alignment and domain prediction of proteins coded by these PSP94-like genes show highly conserved cysteine residues in the PSP94 domain. The data underlying Fig 6B and 6C can be found in S3 Data.

**Fig 7:**
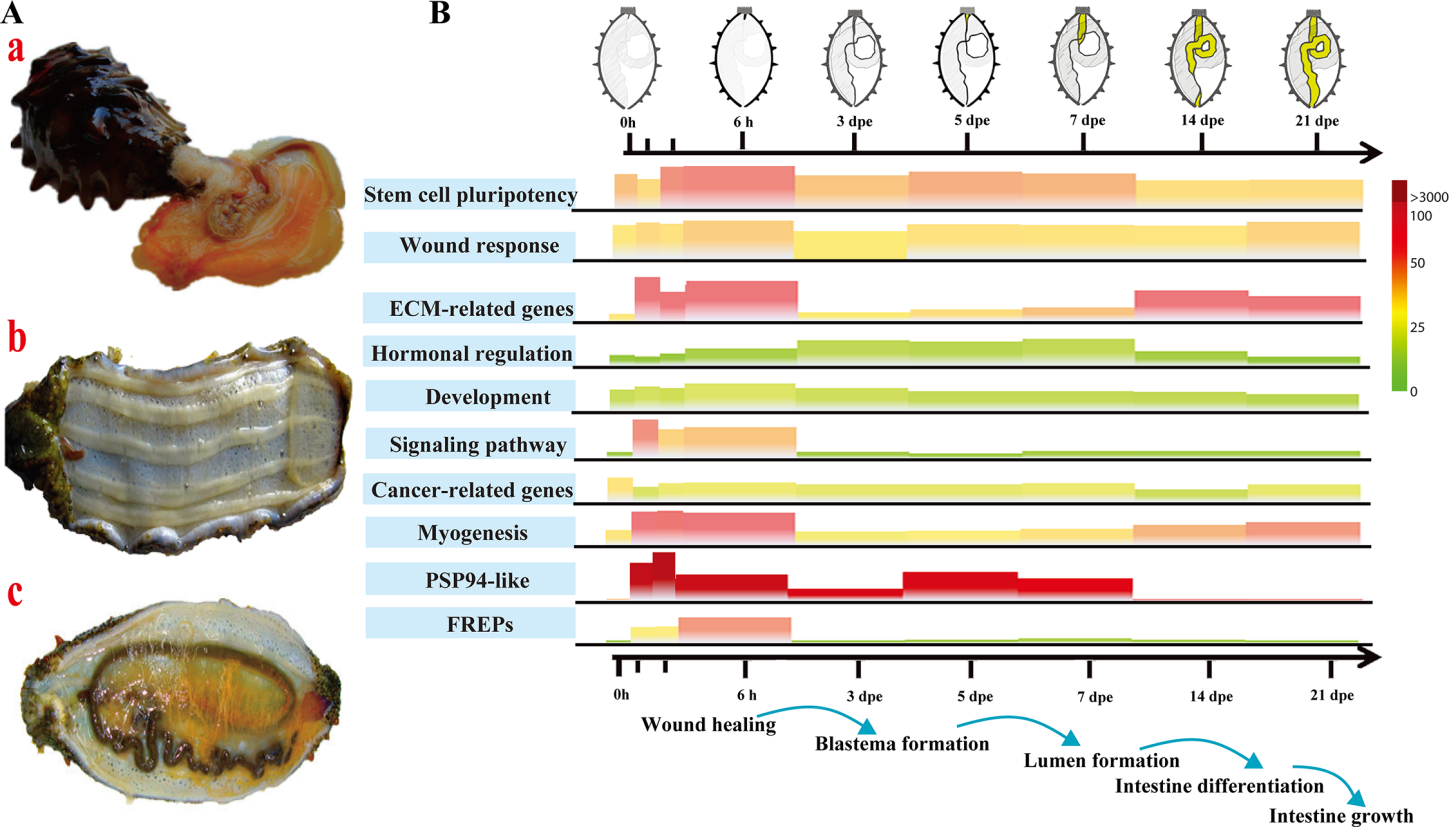
Genes involved in the intestine regeneration of *A*. *japonicus*. (A) Regeneration diagram showing *A*. *japonicus* (a) undergoing evisceration, (b) immediately post evisceration, and (c) after complete recovery. (B) A heatmap showing the expression profile of molecular events applicable to the intestinal regeneration of *A*. *japonicus*. It depicts the molecular events identified in this study in relation to the morphological diversification of regeneration in a diagrammatic sketch. The height and the color of trapezium represent the relative expression level (fragments per kilobase of transcript per million mapped reads [FPKM] value per gene) during the regeneration. High-expressed and low-expressed genes are labeled in red and green, respectively. Extracellular matrix (ECM)-related genes (*collagen* and *fibropellin*), signaling pathways (*Wnt*, bone morphogenetic protein-related genes, and epidermal growth factor-related genes), and myogenesis-related genes (*tubulin*) are activated at the early stage of regeneration (0–3 days post evisceration [dpe]). During the middle stage of regeneration (3–7 dpe), factors related to hormonal regulation are up-regulated. In the late stages of regeneration, ECM-related genes (*tenascin*, *FRAS1*, and *collagen*) and myogenesis-related genes (*actin* and *myosin*) show up-regulated expression. The physiological events happening during regeneration are shown below the *x*-axis. The data underlying Fig 7B can be found in S3 Data. FREP, fibrinogen-related protein.

Evisceration is a defensive strategy shared by many sea cucumbers. In *A*. *japonicus*, it can be induced artificially, and new visceral organs will be regenerated completely in a few weeks, making it an ideal model animal for research on organ regeneration. Gene tandem repeat and whole genome duplication are regarded as primary drivers of genome evolution [44], since they can provide raw material for the generation of novel genes, expansion of gene families, increases in gene dosage, or the development of new functions [45]. In the *A*. *japonicus* genome, 74 clusters with tandem-duplicated genes were found in more than 5 repeats (Fig 6A). When verifying the expression of tandem-duplicated genes from these clusters during visceral regeneration (Fig 7A), we found that genes in 1 cluster located on Scaffold889 showed significant up-regulation by more than 10,000 folds during the early stages (0–3 days post evisceration, dpe) of regeneration (Fig 6B and S14 Fig). In this cluster, the genes were arranged with 11 tandem duplications that had no homologs in other species and contained highly conserved cysteine residues, which suggested that they may belong to a gene family specific to *A*. *japonicus*. Moreover, these genes were specifically expressed in regenerating intestines, with no expression detected in any embryonic or larval stages (S19 Table). Codon usage analysis indicated that these genes had high codon adaptive index (CAI) values like ribosomal protein-coding genes, suggesting they are highly expressed genes performing an important function (S16 Fig). Our corresponding proteomic study confirmed their up-regulation during the same stages (S20 Table). Further analysis on the translated amino acid sequences of these genes showed that they all had a domain that was analogous to the prostatic secretory protein of 94 amino acids (PSP94) based on the topological similarities of the cysteine residues, so we designated them as PSP94-like genes (Fig 6C). It was reported that the PSP94 genes in vertebrates underwent rapid evolution, which resulted in an improvement of their functions in a wide range of biological activities, such as inhibition of tumor growth [46], activation of immunity [47], and mesoderm formation [48]. Taken together, their specific arrangement pattern and distinguished expression profiles strongly suggested that the PSP94-like genes might play a vital role in the regenerative abilities of *A*. *japonicus*.

#### Expanded and clustered fibrinogen-related protein (FREP) genes

Another cluster with 21 tandem-duplicated FREP genes was located on Scaffold75. Many FREPs, like tenascin [49], fibrinogen-like protein [50], angiopoietin [51], and hepassocin [52], have been reported to be involved in the regulation of regeneration. These FREPs showed an obvious genome-wide gene duplication in *A*. *japonicus* and also formed the gene family with the greatest expansion (Fig 1 and S15 Table). Phylogenetic analysis showed that the FREP gene family expanded twice in the *A*. *japonicus* genome, and regeneration-related FREPs were clustered and expanded at the same time (S9 Fig). Immune factors have been reported to play important roles in regeneration [53,54]. They have proved to be critical signals for the activation of cell cycle re-entry during the regeneration of salamander and mammalian liver [54,55]. To investigate whether the FREPs are activated during visceral regeneration, we analyzed their expressions in the transcriptome and proteome data. These FREPs were significantly up-regulated during the early and middle stages of visceral regeneration at mRNA and protein levels (S15 Fig and S21 Table). Collectively, the expansion and tandem duplication of FREPs might underlie the high regenerative potential of the sea cucumber.

#### Conserved regeneration regulation system

Over the past few years, many analyses related to the regeneration process in sea cucumbers have been conducted, providing a list of key candidate genes and a roadmap for future studies of regulatory mechanisms [56–60]. These key candidate genes were classified into several main groups, including developmental genes, extracellular matrix (ECM)-related genes, pluripotency factors, neurogenesis-related genes, and cytoskeletal genes. In the *A*. *japonicus* genome, some regeneration-related factors were found to be exclusively present or expanded, for example, the genes related to ECM-receptor interaction, apoptosis, and adherens junction (S13 and S14 Tables). Our transcriptomic analyses also showed that the visceral regeneration in *A*. *japonicus* was a very complicated process, regulated by a large number of regeneration-related genes, including stem cell pluripotency factors, signaling pathways genes, ECM-related genes, and myogenesis-related genes, and their expression profiles showed differential patterns during regeneration (Fig 7B and S15 Fig). Corresponding proteomic studies confirmed that these factors may play important roles in this process (S22–S24 Tables). Specifically, the most well-characterized set of mammalian pluripotency factors including *Sox2*, *c-Myc*, *Oct4*, and *Klf4*, which can induce transformation of differentiated mammalian fibroblasts into induced pluripotent stem (iPS) cells [61] had corresponding orthologs in *A*. *japonicus*. Among them, the orthologs (*SoxB1*, *Oct1/2/11*, and *Klf1/2/4*) were found to be up-regulated at the early stage post evisceration (0–6 hours) (Fig 7B and S17 Fig). In fact, the key pathways of stem cell pluripotency were considerably conserved as compared to mammals (S18 Fig). Consistent with the phylogenetic position of echinoderms (sister group to chordates), our results show that sea cucumbers share similar characteristics with vertebrates in regard to regeneration, supporting the hypothesis that regenerative mechanisms might be conserved across evolution in the Deuterostomia [62,63]. Meanwhile, some exclusive or significantly expanded genes, such as PSP94-like proteins and FREPs, might play key roles in the remarkable regenerative capacity of sea cucumbers.

### Conclusions and implications

We present a high-quality sea cucumber reference genome from the commercially cultivated species *A*. *japonicus*, generated from Illumina and PacBio platforms. An integrated multiomics approach offered insight into the genome architecture of sea cucumbers and the genetic underpinnings of their unique biological traits. The sea cucumber constitutes a particularly promising model animal for regenerative medicine because of the convenient induction of organ regeneration and the newly available genomic data for *A*. *japonicus*. A genome resource of this completeness and quality also makes an important contribution to holothuroid and echinoderm research. The findings should facilitate our understanding of the requirements for sustainable utilization and effective breeding of echinoderms, in support of the high-value sea cucumber industry.

## Materials and methods

### Animal materials and DNA isolation

The animal material for genome sequencing and assembly was from a male *A*. *japonicus* captured off the coast of Laoshan, Qingdao, China. The sea cucumber was acclimated in sea water at 15 ± 1°C before experiments. Muscle, gonad, and respiratory tree tissues were collected and immediately frozen in liquid nitrogen and stored at −80°C. Genomic DNA was extracted using a TIANamp Marine Animal DNA Kit (TIANGEN, Beijing, China) according to the manufacturer’s instructions.

### Genome sequencing

For Illumina sequencing, short-insert paired-end (PE) (180 bp and 500 bp) and long mate-paired (MP) (5 Kb, 10 Kb, and 20 Kb) DNA libraries were constructed according to the manufacturer’s instructions (Illumina, San Diego, California, US). Sequencing runs for the PE libraries were performed on the Illumina HiSeq2000 platform, and long MP libraries on the HiSeq2500 platform. To obtain long reads to promote genome assembly, Pacific Biosciences RS II (Pacific Biosciences, Menlo Park, California, US) was used as the sequencing platform. Five 10-Kb SMRTbell libraries were prepared and sequenced using the C4 sequencing chemistry and P6 polymerase.

### Genome assembly

In order to get the best assembly results, we tried Short Oligonucleotide Analysis Package de novo assembly tool (SOAPdenovo) [64], *de Bruijn* graph to *Overlap-Layout-Consensus* (DBG2OLC) (https://sourceforge.net/projects/dbg2olc/), FALCON (https://github.com/PacificBiosciences/FALCON), and SMARTdenovo (https://github.com/ruanjue/smartdenovo). The assembly result of SOAPdenovo was fragmented (1,165,887 contigs, N50 1,770 bp, and N90 230 bp). Compared with the SOAPdenovo assembly using only Illumina data, DBG2OLC, FALCON, and SMARTdenovo genome assemblies using PacBio data or a hybrid of PacBio and Illumina data gave more satisfactory results, all with contig N50 values above 110 Kb. This result confirmed the effectiveness of the approaches and the superiority of PacBio long reads for large genome assembly (S2 Table). After comparing the 3 approaches of DBG2OLC, FALCON, and SMARTdenovo, genome assembly by FALCON gave higher continuity (with a contig N50 of 190 Kb) than the other methods.

To test the amount of PacBio data sufficient for *A*. *japonicus* genome assembly, we performed a set of assemblies by FALCON with coverage from 30× to 70× of PacBio data. For all assemblies, default parameters were used. Genome size and N50 of assembled contigs were calculated and used for performance evaluation (S1 Fig**)**. On the basis of these assembly experiments, 70-fold coverage of PacBio data (64 Gb) was sufficient and used for the *A*. *japonicus* genome assembly. The assembled contigs were corrected 8 times with Illumina PE reads using Quiver (https://github.com/PacificBiosciences/GenomicConsensus). Finally, we generated scaffolds and performed gap filling with SSPACE 3.0 (https://www.baseclear.com/genomics/bioinformatics/basetools/SSPACE) using Illumina MP sequencing data.

### Genome assessment

The assembly result was evaluated by remapping high-quality PE reads (180-bp libraries, totaling 128,784,478 paired reads) to scaffolds using Bowtie with parameters of--rdg 3,1--rfg 3,1--gbar 2 [65]. Assembly completeness was examined by mapping 81,639 unigenes from transcriptomes using BLAST (https://blast.ncbi.nlm.nih.gov/Blast.cgi), and the physical coverage of each gene was calculated with SOLAR [66]. The assembled genome was also validated by checking the coverage of 248 conserved core eukaryotic genes using the CEGMA program (version v2.5) [67].

To locate scaffolds on chromosomes, 2 parents and 130 offspring were genotyped, and a high-density linkage map was constructed with Genotyping by Sequencing (GBS) technology [68]. Sequencing depth averaged 50-fold for parents and 10-fold for the 130 offspring. Clean reads were mapped to genome scaffolds by the Burrows-Wheeler Aligner [69]. Variants including SNPs were identified using SAMtools [70]. JoinMap 4.0 was applied for linkage analysis based on a maximum likelihood algorithm [71]. Recombination values were converted to genetic distances in centiMorgans based on the Kosambi mapping function. A total of 71.2% of the genome scaffolds anchored in the linkage map, and Circos64 was used for visualization [72].

To estimate the level of heterozygosity in the genome, we carried out k-mer (where k represents the chosen length of substrings) distribution approximation using simulated heterozygous genome sequences (S2 Fig**)**. Jellyfish were used to calculate k-mer depth distribution [73]. PE reads from short-insert libraries (180 bp) were used for analysis. Adaptor and low-quality reads were trimmed using the NGS QC Toolkit (v2.3.3) [74]. Reads for 26.01 Gb of clean data were split into k-mers, and k-mer depth was computed to obtain depth distribution. Two peaks of k-mer depth 14 and 28 were observed in 17-mer analysis. We found that the k-mer distribution was fitted best by a simulated k-mer distribution with 2% heterozygosity (S3 Fig). To obtain an exact heterozygous rate, we used the software gce to analyze k-mer frequency data with parameters of -c unique_depth -H 1 (ftp://ftp.genomics.org.cn/pub/gce). We used gce to estimate the k-mer heterozygous ratio (KHR) based on the following formula: KHR = a1 / 2 / (2 − a1 / 2). We used the formula base heterozygous ratio (BHR) = KHR / Kmer (assuming each SNP caused K new k-mers) for BHR. An exact heterozygous rate of 1.59% was estimated, which was close to the evaluation by simulated k-mer distribution. We also investigated the level of heterozygosity by mapping all reads back to the assembled genome using BWA with default parameters. For reads with multiple mapping positions, only the single best hit was retained. SNPs and INDELs were called based on alignment results with SAMtools [70]. A number of SNPs (5,379,554) and INDELs (486,341) were detected on the assembly genome, suggesting heterozygosity of about 0.73%. Because the part of the genome with the lowest heterozygosity had the best assembly, 0.73% was considered an underestimation due to sampling bias.

### Repeat sequence analysis

RepeatModeler (http://www.repeatmasker.org/RepeatModeler/) was used for repeat family identification in a de novo approach. RepeatMasker was used to identify transposable elements by aligning genome sequences against RepBase (RepBase21.04) and a local library generated by RepeatModeler with default parameters. Tandem repeats were analyzed with the Tandem Repeats Finder (TRF) program [75]. Transposable elements (TEs) in the *A*. *japonicus* genome were discovered by a combination of de novo-based and homology-based approaches.

A local repeat library of 1,507 sequences was produced by RepeatModeler and was used for the following prediction [76]: All major classes of TEs were summarized and compared with *S*. *purpuratus* (S7 Table). A total of 210.87 Mb (26.20%) of the assembled genome was predicted to be TEs, slightly less than *S*. *purpuratus* (32.67%). Of these, 18.93% were classified as unknown repeats. Among the 7.10% annotated repeat families, retrotransposable element Long Interspersed Nuclear Elements (LINEs) (2.31%) and DNA transposons (3.20%) were 2 major TEs discovered in the assembled genome that were similar to *S*. *purpuratus*. However, *S*. *purpuratus* has more DNA transposable elements (8.36%) than *A*. *japonicus* (S7 Table and S4 Fig).

### Noncoding RNA prediction

Noncoding RNA genes were predicted for repeat-masked genome by sequence- and structure-based alignments with the Rfam noncoding RNA database (http://xfam.org/) with an E-value cutoff at 0.01, using Infernal [77]. Specifically, for miRNA identification, small RNA sequencing data were downloaded from the SRA database of NCBI, including sRNAs from respiratory tree (SRX878425), tubefoot (SRX642039), and longitudinal muscle (SRX878452). Adaptor and primer sequences were trimmed, and low-quality sequences removed. Clean sRNA reads were compared with the Rfam database (http://xfam.org/) to exclude noncoding RNAs other than miRNAs. The remaining sRNA reads were subjected to miRNA identification by mapping to predicted pre-miRNA structures in the *A*. *japonicus* genome using miRDeep2 [78] and miReap (http://sourceforge.net/projects/mireap/). Finally, novel and conserved miRNAs were classified by searching against the miRBase (http://mirbase.org/).

### Gene prediction and function annotation

Three approaches were used to predict protein-coding genes: homology-based predictions, de novo predictions, and transcriptome-based predictions. Homologous proteins from 8 known whole genome sequences *Homo sapiens*, *Danio rerio*, *B*. *floridae*, *S*. *kowalevskii*, *S*. *purpuratus*, *Daphnia pulex*, *C*. *gigas*, and *Hydra vulgaris *were used for alignment to the repeat-masked *A*. *japonicus* genome using Exonerate (version 2.2.0) [79]. Genewise (version 2.2.0) was used to generate gene structures based on homology alignments of proteins to the genome [80,81]. For ab initio gene prediction, we used Augustus (version 2.5.5), Genescan (version 1.0), GlimmerHMM (version 3.0.1), and SNAP15 to predict coding genes. RNA-Seq data were used to improve gene annotation and mapped to the genome using Tophat (version 2.0.8) [82]. Cufflinks (version 2.1.1) (http://cole-trapnell-lab.github.io/cufflinks/) was used to identify spliced transcripts in gene models [83]. All gene evidence predicted from the 3 approaches was combined by EVM into a weighted and nonredundant consensus of gene structures [84]. Gene models generated by EVM were filtered according to the following criteria: coding region lengths less than 150 bp and values of reads per kilobase of exon model per million mapped reads (fragments per kilobase of transcript per million mapped reads [FPKM]) < 5 when a predicted gene supported by ab initio methods only hit with the uniref 90 database [85]. For gene functional prediction, NCBI nr and the SwissProt database were used for gene blasts. All predicted genes were blasted against the 2 databases using BLASTP (E-value ≤ 1E-10).

### Gene family analysis

To understand the evolutionary relationship of *A*. *japonicus* with other metazoans, we performed systematic gene comparisons. Full protein-coding genes of 17 genomes, including human (*H*. *sapiens*), lizard (*Anolis carolinensis*), turtle (*Chrysemys picta*), frog (*Xenopus tropicalis*), zebrafish (*D*. *rerio*), amphioxus (*B*. *floridae*), acorn worm (*S*. *kowalevskii*), sea urchin (*S*. *purpuratus*), sea star (*A*. *planci*), sea cucumber (*A*. *japonicus*), water flea (*D*. *pulex*), leech (*Helobdella robusta*), oyster (*C*. *gigas*), octopus (*Octopus bimaculoides*), sea anemone (*Nematostella vectensis*), trichoplax (*Trichoplax adhaerens*), and sponge (*Amphimedon queenslandica*), were used for comparisons.

For greater insight into the evolutionary dynamics of the genes, we determined the expansion and contraction of the gene ortholog clusters among these 17 species. We used CAFE software for computational analysis of gene family evolution [86] and defined expansion and contraction by comparing cluster size differences between ancestors and each current species. Extinction and evolution of gene families were processed by CAFE, using a random birth and death process model to identify gene gain and loss along each lineage of the RAxML tree (Fig 2). For all species, expanded and contracted gene families (compared to ancestors) were compared with *A*. *japonicus* to identify gene families that expanded or contracted only in the sea cucumber.

For genes exclusively present and gene families specifically expanded in *A*. *japonicus*, we conducted gene ontology (GO) enrichment analysis and Kyoto Encyclopedia of Genes and Genomes (KEGG) pathway enrichment analysis using blast2go and KAAS [87,88]. Using Omicshare CloudTools (http://www.omicshare.com/tools/?l=en-us), enriched GO terms and KEGG Orthology (KO) terms were calculated relative to the background of full protein-coding genes. Among the 30,350 protein-coding genes of *A*. *japonicus*, many-to-many orthologs (15.38%), patchy orthologs (36.44%), and species-specific genes (43.17%) were 3 major gene model groups (Fig 2C). Echinoderm-specific orthologs (1.94%) were also detected in the *A*. *japonicus* genome. GO enrichment analyses of the *A*. *japonicus*-specific genes and significantly expanded gene families suggested that most genes were associated with GO terms in cellular process, metabolic process, and binding and catalytic activity (S6 and S8 Figs). Based on the full protein-coding genes, *A*. *japonicus*-specific genes were predominately enriched in GO terms related to signal recognition and immunity (S12 Table and S8 Fig). Similar results were found for GO enrichment analysis of significantly expanded gene families relative to full genes (S13 Table, S8 Fig). KEGG pathway enrichment analyses indicated that *A*. *japonicus*-specific genes and gained genes were also enriched in pathways related to signal recognition and immunity (S14 and S15 Tables, S8 Fig).

### Phylogenetic tree construction

Peptide sequences were clustered by the Markov clustering program orthoMCL [89]. These sequences were also searched against the nr database by an all-versus-all BLASTP with threshold E ≤ 1E-05 and then clustered by MCL with an inflation value of 1.5. A total of 43 single-copy orthologous genes were clustered among 17 genomes. Ortholog alignments were produced using MUSCLE (v3.6) and concatenated into a single multiple-sequence alignment by an in-house Perl script. A neighbor-joining phylogeny was reconstructed using MEGA (v5) [90].

To examine the phylogeny of sea cucumbers, we used a maximum likelihood (ML) method for genome-wide phylogenetic analysis based on single-copy genes from the 10 deuterostome and 7 nondeuterostome genomes. Based on the gene clustering results from orthoMCL, 49,351 gene families were collected from the 17 species. Among them, 43 single-copy genes were used for phylogenetic tree construction. To understand the relationship of *A*. *japonicus* to other echinoderms, we constructed another phylogenetic tree of 5 echinoderms based on orthologous genes. To extend the taxonomic sampling, gene families were surveyed in transcriptome datasets in *F*. *serratissima*, *Patiria miniata*, and *A*. *filiformis* (NCBI SRA database accession numbers SRR2454338, SRR573710, SRR573709, SRR573708, SRR573706, SRR573707, SRR573705, SRR573675, SRR1523743, SRR1533125, SRR794587, SRR794568, SRR789489, and SRR3097584). Transcriptome data for the 3 echinoderms were assembled into unigenes using Trinity and cap3 with default parameters [91,92]. Single-copy gene families were extracted from the full genes of *S*. *kowalevskii* (outgroup), *A*. *japonicus*, and *S*. *purpuratus*. For each gene family, protein sequences from all represented sequenced genomes were searched using BLASTP (E-value cutoff 1.00E-10) against unigenes from each species. The unigene with the best score was translated as the longest open reading frame (ORF) in the frame detected by BLASTP. Gene clustering analysis was performed on the unigenes. Finally, 2,066 orthologous genes were obtained for constructing a phylogenetic tree using the ML method. For ML tree construction, sequence alignments were performed using MUSCLE 3.6 [93]. The substitution models that best fit observed alignment data were estimated using the program jModelTest 2 [94]. Using PhyML [95], we performed ML analysis with the substitution model WAG + gamma + Inv, and 1,000 bootstraps were conducted to produce the branch support values.

### Divergence time estimation

Molecular clocks and divergence time were estimated by combining r8s and RAxML programs [96,97]. Maximum likelihood phylogeny and branch lengths were obtained by RaxML with 1,000 bootstrap replicates. The birth-death model with fossil calibrations was used for Bayesian estimation of species divergence times [94,98–105]. Fossil-derived timescales and evolutionary history were obtained from TIMETREE [106]. The divergence time of TEs was calculated using RepeatMasker (http://www.repeatmasker.org/).

### Morphological evolution analyses

#### Pharyngeal gill slits gene cluster

A conserved deuterostome-specific pharyngeal gene cluster was discovered to be important for pharyngeal gill slits [24]. The gene cluster was also located on a single scaffold in *A*. *japonicus*, but with altered gene cluster order. Generally, *nkx2*.*1*-*nkx2*.*2* and *pax1/9*-*slc25A21* were the 2 most conserved subclusters in the gene cluster, but both were interrupted in *A*. *japonicus*. The homologous gene *slc25A29* was far from *pax1/9*, while *nkx2*.*1* was in another scaffold. The *pax1/9* gene is important for formation of gill slits via modulation of *six1/2* and *tbx1/10* expression [107]. In *A*. *japonicus*, *six1* was a neighbor to *six6* and *six4*, which form a gene cluster similar to that of chordates and hemichordates (S6 Fig).

#### Notochord formation-related gene families

We searched the *A*. *japonicus* genome for genes involved in notochord formation (Fig 3). To determine the copy number of *FGF* genes in the *A*. *japonicus* genome, we screened the *A*. *japonicus* transcriptome data using full FGF genes from the other 16 species. Only a single unigene was homologous to an *FGF* gene.

*foxE* is specifically expressed in the buccal and pharyngeal regions and the stomochord region, and it is a marker gene for stomochord formation [30]. We compared *fox* genes of *S*. *kowalevskii* against *A*. *japonicus* transcriptome unigenes. Several unigenes were homologous to *fox* genes; however, no unigenes belonged to *foxE*. To further test if foxE was absent in echinoderms, we compared *S*. *kowalevskii fox* genes against the draft assembly of other echinoderms from NCBI, including sea urchin *Lytechinus variegates* (AGCV00000000.2), sea cucumber *Parastichopus parvimensis* (JXUT00000000.1), brittle star *Ophiothrix spiculata* (JXSR00000000.1), and sea star *P*. *miniata* (AKZP00000000.1). No *foxE* gene was detected in these genomes (S16 Table).

#### Homeobox gene families

To obtain *A*. *japonicus* homeobox genes, we screened the genome against a homeobox gene database (http://homeodb.zoo.ox.ac.uk/) and identified 108 homeobox genes. These genes belonged to the classes ANTP-HOXL (18), ANTP-NKL (26), PRD (28), LIM (9), POU (9), HNF (1), SINE (3), TALE (7), CUT (3), PROS (1), ZF (2), and CERS (1) (S17 Table). We found a Hox gene cluster containing 10 conserved Hox genes in the *A*. *japonicus* genome. The genes were *Hox1*, *Hox2*, *Hox3*, *Hox5*, *Hox7*, *Hox8*, *Hox9/10*, *Hox11/13a*, *Hox11/13b*, and *Hox11/13c*. *Hox4* and *Hox6* were not found.

#### Skeletal development genes

We compared the genes of skeletogenic regulatory systems in sea cucumbers and sea urchins and found that biomineralization genes were significantly different between the 2 echinoderms. There are 31 biomineralization proteins in sea urchins that are important for osteogenesis [39,108]; however, only 7 homologous biomineralization genes were identified in the *A*. *japonicus* genome (S19 Table). Furthermore, we searched for these 31 genes in the sea star *A*. *planci* (NCB Bioproject PRJDB3175) and the hemichordate *S*. *kowalevskii* (NCB Bioproject PRJNA12887) and listed the numbers of the genes in the 4 animals.

To compare the expression of the biomineralization genes of sea urchins and sea cucumbers, we collected expression data on the 31 biomineralization proteins of *S*. *purpuratus* from the “Quantitative Developmental Transcriptomes of *S*. *purpuratus*” in Echinobase (http://www.echinobase.org:3838/quantdev/), which includes 4 development stages: fertilized eggs (10 hours), blastula (24 hours), gastrula (48 hours), and pluteus (72 hours). The corresponding expression information for 4 biomineralization genes was obtained from *A*. *japonicus* developmental transcriptomes. Using *tubulin* expression as the control, the biomineralization genes showed significantly lower expression in *A*. *japonicus* than *S*. *purpuratus* (Fig 3F).

### Visceral regeneration

In order to better understand the regeneration mechanism, we conducted a set of genomic and multiomic analyses of intestinal regeneration in *A*. *japonicus*. Adults of *A*. *japonicus* (100–120 g) were collected from the coast of Qingdao, Shandong Province. They were held in a laboratory for 1 week in sea water at 15–17°C and fed once a day. Evisceration was induced by injecting about 2 mL 0.35 molL^-1^ KCl into the coelom. Eleven individuals per stage were sampled at 0.5 hours, 2 hours, 6 hours, 3 dpe, 5 dpe, 7 dpe, 14 dpe, and 21 dpe, and intestinal tissues were collected for RNA isolation; noneviscerated sea cucumbers served as controls. Dissected intestines were frozen in liquid nitrogen and stored at −80°C until RNA extraction.

Total RNA was extracted using the TRIzol extraction method (Thermo Fischer Scientific, Germany) according to the manufacturer’s protocol. Poly-A mRNA was isolated from 40 μg total RNA per sample using oligo-dT-coupled beads and sheared. Isolated RNA samples were used for first-strand cDNA synthesis using random hexamers and Superscript II reverse transcriptase. After end repair and addition of a 3′-dA overhang, cDNA was ligated to an Illumina paired-end adapter oligo mix and size-selected by gel purification to enrich for approximately 200 bp fragments. After 16 PCR cycles, transcriptomes of the entire intestinal regeneration process of 9 stages were sequenced using Illumina HiSeq-2000 and the PE end sequencing module. These stages corresponded to 6 key phases of intestinal regeneration: early response (0 to 6 hours post evisceration), wound healing (6 hours to 3 dpe), blastema formation (3 to 7 dpe), lumen formation (7 to 14 dpe), intestinal differentiation (14 to 21 dpe), and growth (21 dpe) [57,109]. RNA expression analysis was based on the predicted genes of *A*. *japonicus* genome. Tophat was used to map mRNA reads to the genome, and Cufflinks was used to calculate expected FPKM as expression values for each transcript.

Corresponding proteomic studies (3, 5, 7, 14, and 21 dpe) of intestinal regeneration were analyzed using isobaric tags for relative and absolute quantitation (iTRAQ) coupled with mass spectrometry (MS). Total protein was taken from sample solutions, and protein was digested with Trypsin Gold (Promega, Madison, Wisconsin, US) at a protein:trypsin ratio of 30:1 at 37°C for 16 hours. After digestion, peptides were dried by vacuum centrifugation, reconstituted in 0.5 mol/L TEAB, and processed according to the manufacturer’s protocol for 8-plex iTRAQ reagent (Applied Biosystems, Foster City, California, US). The LC-ESI-MS/MS analysis was based on Triple TOF 5600 (AB SCIEX, Ontario, Canada). Raw data files were acquired from Orbitrap and converted into MGF files by Proteome Discoverer 1.2 (PD 1.2, Thermo Fischer Scientific). Protein identification used the Mascot search engine (Matrix Science, London, United Kingdom).

## Supporting information

S1 Data Noncoding RNAs in the *A*. *japonicus* genome. (XLSX)

S2 Data Some genes related to nervous system development. (XLSX)

S3 Data Supplementary data for the figures. (XLSX)

S1 Fig Genome size and N50 distribution of the genome assembled with different coverage of PacBio data. (EPS)

S2 Fig Sequencing coverage distribution of the bases and genome fragments. (A) Sequencing depth distribution of the bases throughout the genome. (B) Plot of guanine-cytosine (GC) content against the average sequencing depths of 50 Kb fragments. The average sequencing depth was calculated on each 50 Kb nonoverlapping sliding window. The clustered scatter points indicate that no heterozygous sequences were found in the assembled genome.
(TIF)

S3 Fig Distribution profiles of unique K-mer counts in the raw sequencing reads of *A*. *japonicus* and simulated heterozygous genome sequences. Target indicates the genome of *A*. *japonicus*; H0.01–H0.06 indicate the simulated genome with heterozygosity of 1%–6%, respectively.
(EPS)

S4 Fig Age distribution of repeats in the *A*. *japonicus* and *S*. *purpuratus* genomes. The substitution rates were calculated between the genomic and the repeat consensus sequences. (A) The 3 transposable element expansion peaks were found in the *A*. *japonicus* genome, and (B) 2 expansion peaks were found in the *S*. *purpuratus* genome.
(EPS)

S5 Fig Gene structure comparison among *A*. *japonicus*, *S*. *purpuratus* and *A. planci*. (A) Exon length distribution. (B) Exon number distribution. (C) Intron length distribution. (D) Gene length distribution.
(EPS)

S6 Fig Phylogenetic relationship of 5 species of echinoderms. The maximum-likelihood tree was obtained with a supermatrix of 305,524 amino-acid residues gathered from 2,066 orthologous genes in 5 species of echinoderms and 1 outgroup species, *S*. *kowalevskii*. Image credits: Jerry Kirkhart; authors' own; public domain; Freshwater and Marine Image Bank; National Oceanic and Atmospheric Administration; Encyclopædia Britannica.
(EPS)

S7 Fig Gene family distribution of cytochrome P450 among 3 species of echinoderms. The numbers in the heatmap are gene copies of each class of cytochrome P450. Genes encoding cytochrome P450 are expanded in *A*. *japonicus* and *S*. *purpuratus*, except cytochrome P450 class 3A24 and 4V2.
(EPS)

S8 Fig Gene ontology (GO) and Kyoto Encyclopedia of Genes and Genomes (KEGG) pathway enrichment analysis of the genes exclusively present or expanded in the *A*. *japonicus* genome. (A) Distribution of the specific genes in each GO term. (B) Distribution of the expanded genes in each GO term. Most genes are enriched in GO terms of cellular process, metabolic process, binding, and catalytic activity. (C) KEGG pathway enrichment analysis of the genes exclusively present in the *A*. *japonicus* genome. (D) KEGG pathway enrichment analysis of the expanded genes in the *A*. *japonicus* genome.
(EPS)

S9 Fig Phylogenetic tree of fibrinogen-related proteins (FREPs) in *A*. *japonicus* and other species. (A) The maximum-likelihood phylogenetic tree of FREPs in *A*. *japonicus* and other species. The genes of *A*. *japonicus* are shown in red, and the genes of other species are shown in different colors: *Homo sapiens* (green); *Anolis carolinensis* (purple); *Chrysemys picta* (khaki); *Danio rerio* (blue); *Xenopus tropicalis* (gray); *Ciona intestinalis* (black); *Branchiostoma floridae* (royal purple); *Saccoglossus kowalevskii* (dark green); *Strongylocentrotus purpuratus* (pink); *Crassostrea gigas* (cyan); *Nematostella vectensis* (yellow); *Helobdella robusta* (buff); *Drosophila melanogaster* (tan); and *Octopus bimaculoides* (orange). (B) Relative evolutionary time of different FREPs in *A*. *japonicus*.
(EPS)

S10 Fig Linkage and order of the *six* gene cluster across metazoans. *six6*, *six1*, and *six4* are 3 genes of the *six* gene cluster that showed consistent synteny across deuterostomes (green background), whereas they were incomplete and distributed in different scaffolds within nondeuterostomes (blue background).
(TIF)

S11 Fig Expression level of *brachury* and *FGF9* at different development stages. *brachury* and *FGF9* showed high expression levels at the stages of gastrula, doliolaria, and pentactula. Image credits: Robert Michniewicz; Kobie Mercury-Clarke; Narbonne P, Simpson D, Gurdon J; Vector Open Stock; Lars Simonsen; Freshwater and Marine Image Bank; Encyclopædia Britannica; public domain; Jerry Kirkhart; Authors' own; Martin Cooper; public domain; public domain; Cnidaria; Freshwater and Marine Image Bank; Maja Adamska; Johny Ha; Gewin V; Eitel M, Osigus H-J, DeSalle R, Schierwater B.
(EPS)

S12 Fig In situ hybridization of *brachury* at different development stages of *A*. *japonicus*. *brachury* was specifically expressed around the mouth, which was similar to the expression pattern of the gene from other echinoderms.
(EPS)

S13 Fig Expressions of gill-slit related genes at different development stages of *A*. *japonicus*. *Nkx2*.*1*, *FoxA*, and *Pax1/9* were highly expressed at the gastrula and doliolaria stages.
(EPS)

S14 Fig Validation of the expression level of 5 prostatic secretory protein of 94 amino acids (PSP94)-like genes by quantitative PCR (qPCR) during the intestine regeneration process of *A*. *japonicus*. The data are expressed as mean ± SD after normalization.
(TIF)

S15 Fig Details of the gene categories related to intestine regeneration in *A*. *japonicus*. The information provided in the figure for each category is the expression profile in each regeneration stage.
(EPS)

S16 Fig Codon usage analysis of the protein-coding genes of *A. japonicus*. (A) Plot of the second important axis (Axis2) after correspondence analysis against the codon adaptive index (CAI). A strong positive correlation between Axis2 and CAI indicates that gene expression level is one of the major factors affecting codon usage. (B) Plot of CAI against the effective number of codons (Nc). Blue circles stand for the genes encoding ribosomal proteins, which are commonly considered as highly expressed proteins. Red circles stand for 11 prostatic secretory protein of 94 amino acids (PSP94)-like genes. Like ribosomal proteins, PSP94-like genes showed high CAI and Nc values, suggesting that PSP94-like genes are also highly expressed genes.
(TIF)

S17 Fig Heatmap showing the expression profile of 4 orthologs of key pluripotency factors (*Sox2*, *c-Myc*, *Oct4*, and *Klf4*) during intestinal regeneration in *A*. *japonicus*. The orthologs of the 4 key pluripotency factors in *A*. *japonicus* were *SoxB1* (AJAP16673), *Myc* (AJAP11986), *Oct1/2/11* (AJAP10188), and *Klf1/2/4* (AJAP08993). Color illustrates the fold changes of gene expression level between adjacent regeneration stages. Red represents up-regulation, and green represents down-regulation.
(TIF)

S18 Fig Analogy with the known pluripotency pathways from mammalian genomes. Factors that had potential homologues in *A*. *japonicus* are shown in a green box, whereas a red box means the homologues could not be found in the *A*. *japonicus* genome. The image was modified from the signaling pathways regulating pluripotency of stem cell (ko04550) from the Kyoto Encyclopedia of Genes and Genomes (KEGG) and the pathway of embryonic stem cell pluripotency in mouse from Qiagen.
(EPS)

S1 Table Summary of Illumina and PacBio sequencing data. (XLSX)

S2 Table Quality statistics of PacBio sequencing data. (XLSX)

S3 Table Genome assembly of *A*. *japonicus* using 4 different software programs: Short Oligonucleotide Analysis Package de novo assembly tool (SOAPdenovo), *de Bruijn* graph to *Overlap-Layout-Consensus* (DBG2OLC), Fast Alignment and Consensus for Assembly (FALCON), and SMARTdenovo. (XLSX)

S4 Table Assembly statistics of published marine invertebrate genomes. (XLSX)

S5 Table Summary of the final genome assembly. (XLSX)

S6 Table Gene region coverage assessed based on transcriptome. (XLSX)

S7 Table Gene region coverage assessed by core eukaryotic genes in Core Eukaryotic Genes Mapping Approach (CEGMA). (XLSX)

S8 Table Summary of the transposable elements. (XLSX)

S9 Table General features of *A*. *japonicus* and *S*. *purpuratus* protein-coding genes. (XLSX)

S10 Table Gene ontology (GO) enrichment analysis of echinoderm-specific gene families. (XLSX)

S11 Table Gene ontology (GO) enrichment of the genes exclusively present in *A*. *japonicus*. The gene ratios in each GO term are compared between the specific and full protein-coding genes of the genome. The GO terms with a light green background are associated with immunity, and those with a yellow background are associated with signal recognition.
(XLSX)

S12 Table Gene ontology (GO) enrichment of the expanded gene families in *A*. *japonicus*. The gene ratios in each GO term are compared between the significantly expanded and full protein-coding genes of the genome.
(XLSX)

S13 Table Kyoto Encyclopedia of Genes and Genomes (KEGG) pathway enrichment of the genes exclusively present in *A*. *japonicus*. The gene ratios in each KEGG Orthology (KO) term are compared between the specific and full protein-coding genes of the genome. The KO terms with a light green background are associated with immunity, and those with a yellow or blue background are associated with signal recognition and regeneration, respectively.
(XLSX)

S14 Table Kyoto Encyclopedia of Genes and Genomes (KEGG) pathway enrichment of the expanded gene families in *A*. *japonicus*. The gene ratios in each KEGG Orthology (KO) term are compared between the significantly expanded and full protein-coding genes of the genome. The KO terms with a light green background are associated with immunity, and those with a yellow or blue background are associated with signal recognition and regeneration, respectively.
(XLSX)

S15 Table Top 8 significantly expanded gene families in *A*. *japonicus*. Acar, *Anolis carolinensis*; Cpic, *Chrysemys picta*; Hsap, *Homo sapiens*; Xtro, *Xenopus tropicalis*; Drer, *Danio rerio*; Bflo, *Branchiostoma floridae*; Skow, *Saccoglossus kowalevskii*; Spur, *Strongylocentrotus purpuratus*; Ajap, *A*. *japonicus*; Dpul, *Daphnia pulex*; Hrob, *Helobdella robusta*; Cgig, *Crassostrea gigas*; Obim, *Octopus bimaculoides*; Nvec, *Nematostella vectensis*; Tadh, *Trichoplax adhaerens*; and Aque, *Amphimedon queenslandica*.
(XLSX)

S16 Table Fox genes in 4 echinoderm genomes. The draft scaffolds of 4 echinoderm genomes were blasted against the Fox gene family, and the homologous regions were extracted to blast against the nr database. Gene names indicate the best hit gene from the nr database.
(XLSX)

S17 Table Homeobox gene family of *A*. *japonicus*. (XLSX)

S18 Table Main signal pathway and genes involved in regulation of skeletogenesis in sea urchin (*S*. *purpuratus*) and sea cucumber (*A*. *japonicus*). (XLSX)

S19 Table Expression level of prostatic secretory protein of 94 amino acids (PSP94)-like genes at different development stages in *A*. *japonicus*. (XLSX)

S20 Table Detail of 7 prostatic secretory protein of 94 amino acids (PSP94)-like proteins found in the proteome result. The up-regulated folds between 0–3 days post evisceration (dpe) are shown.
(XLSX)

S21 Table Top 10 up-regulated proteins between 7 and 14 days after intestine regeneration in *A*. *japonicus*. The fibrinogen-related proteins (FREPs) are shown in red.
(XLSX)

S22 Table Summary of proteomic results from isobaric tags for relative and absolute quantitation (iTRAQ). (XLSX)

S23 Table Statistics of differential protein (fold changes ≥ 1.2 and *P* ≤ 0.05) during visceral regeneration in *A*. *japonicus*. (XLSX)

S24 Table The significantly up-regulated regeneration-related proteins found in the corresponding proteomic studies (3, 5, 7, 14, and 21 days post evisceration [dpe]) during the intestinal regeneration process in *A*. *japonicus*. (XLSX)

## Abbreviations

CAI: codon adaptive index
CARD: caspase recruitment domain
CCHC: CysCysHisCys
CEGMA: Core Eukaryotic Genes Mapping Approach
CYP2: cytochrome P450 family 2
DBG2OLC: *de Bruijn* graph to *Overlap-Layout-Consensus*
dpe: days post evisceration
ECM: extracellular matrix
FALCON: Fast Alignment and Consensus for Assembly
FGF: fibroblast growth factor
FPKM: fragments per kilobase of transcript per million mapped reads
FREP: fibrinogen-related protein
GC content: guanine-cytosine content
GO: gene ontology
iPS: induced pluripotent stem
ISH: in-situ hybridization
iTRAQ: isobaric tags for relative and absolute quantitation
KEGG: Kyoto Encyclopedia of Genes and Genomes
KO: KEGG Orthology
miRNA: microRNA
NLR: nucleotide-binding oligomerization domain-like receptors
PMC: primary mesenchyme cell
PSP94: prostatic secretory protein of 94 amino acids
qPCR: quantitative PCR
SMRT: Single Molecule Real Time
SOAPdenovo: Short Oligonucleotide Analysis Package de novo assembly tool
snoRNA: small nucleolar RNA
snRNA: small nuclear RNA
TAI: translocation and inversion
