# Supplementary figures and images for: The sea cucumber genome provides insights into morphological evolution and visceral regeneration
Source: PLoS Biol. 2017 Oct 12;15(10):e2003790. doi: 10.1371/journal.pbio.2003790 (PMC5638244; doi:10.1371/journal.pbio.2003790)

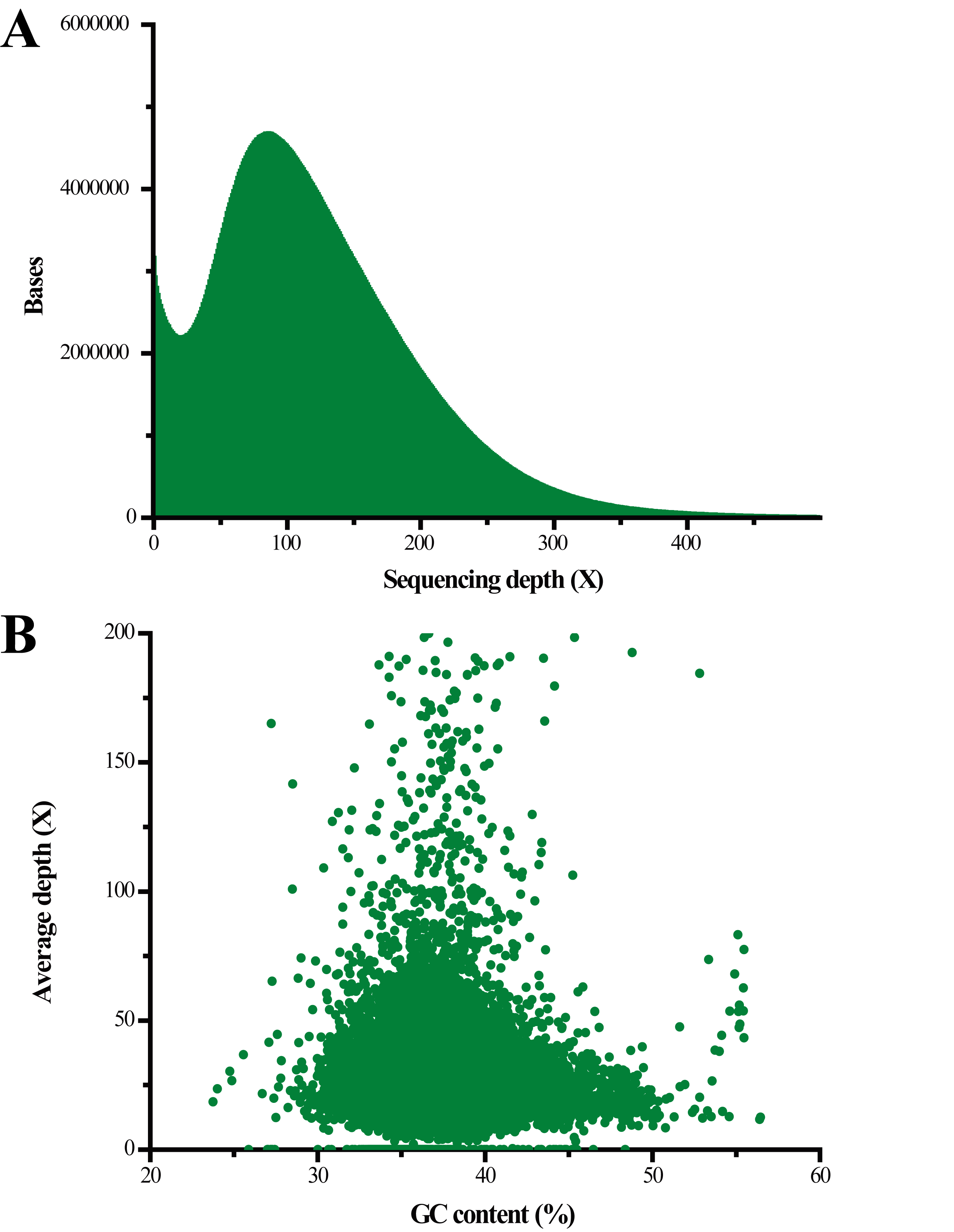

Supplement: S2 Fig — (A) Sequencing depth distribution of the bases throughout the genome. (B) Plot of guanine-cytosine (GC) content against the average sequencing depths of 50 Kb fragments. The average sequencing depth was calculated on each 50 Kb nonoverlapping sliding window. The clustered scatter points indicate that no heterozygous sequences were found in the assembled genome. (TIF) [file pbio.2003790.s005.tif]

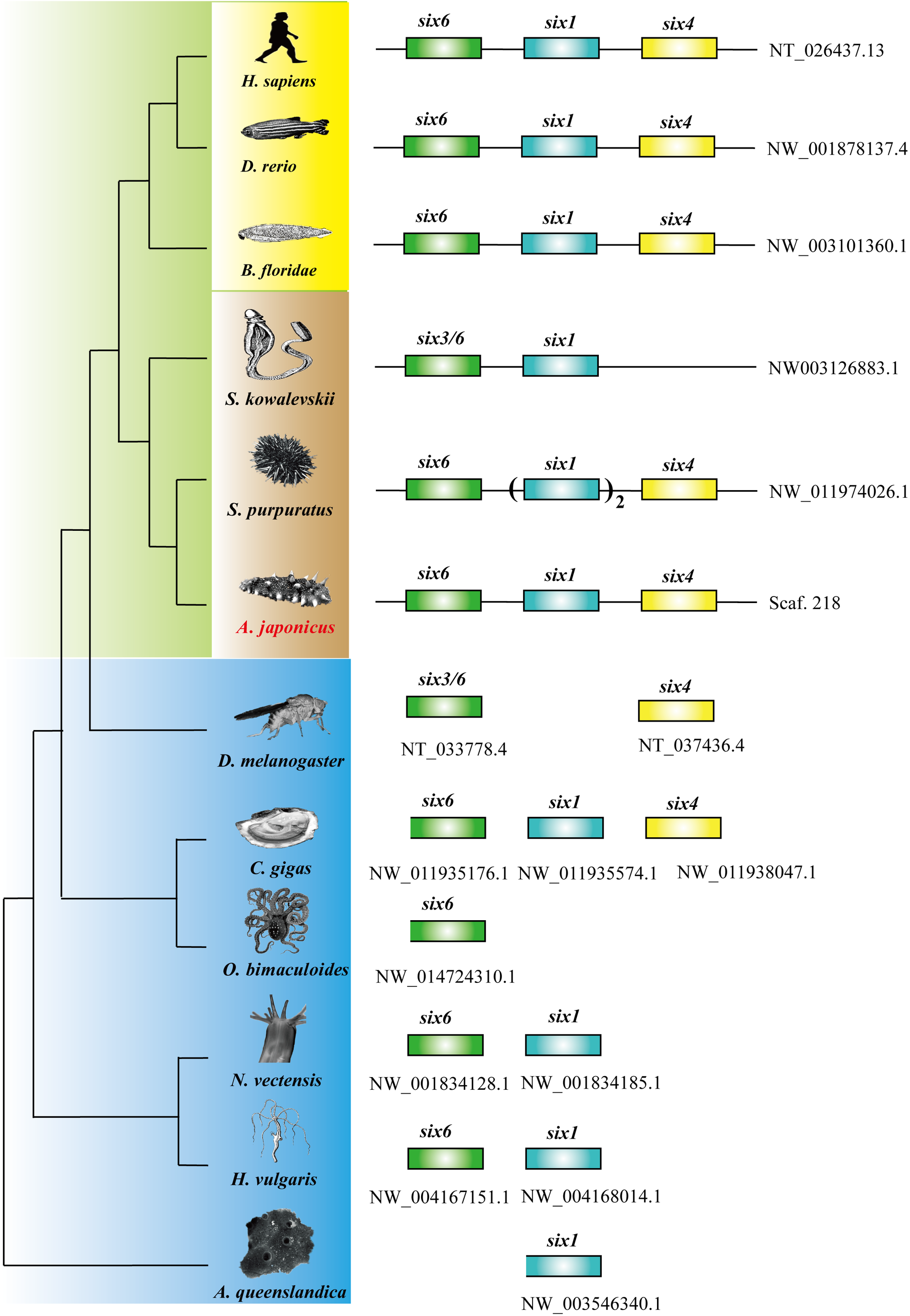

Supplement: S10 Fig — six6, six1, and six4 are 3 genes of the six gene cluster that showed consistent synteny across deuterostomes (green background), whereas they were incomplete and distributed in different scaffolds within nondeuterostomes (blue background). (TIF) [file pbio.2003790.s013.tif]

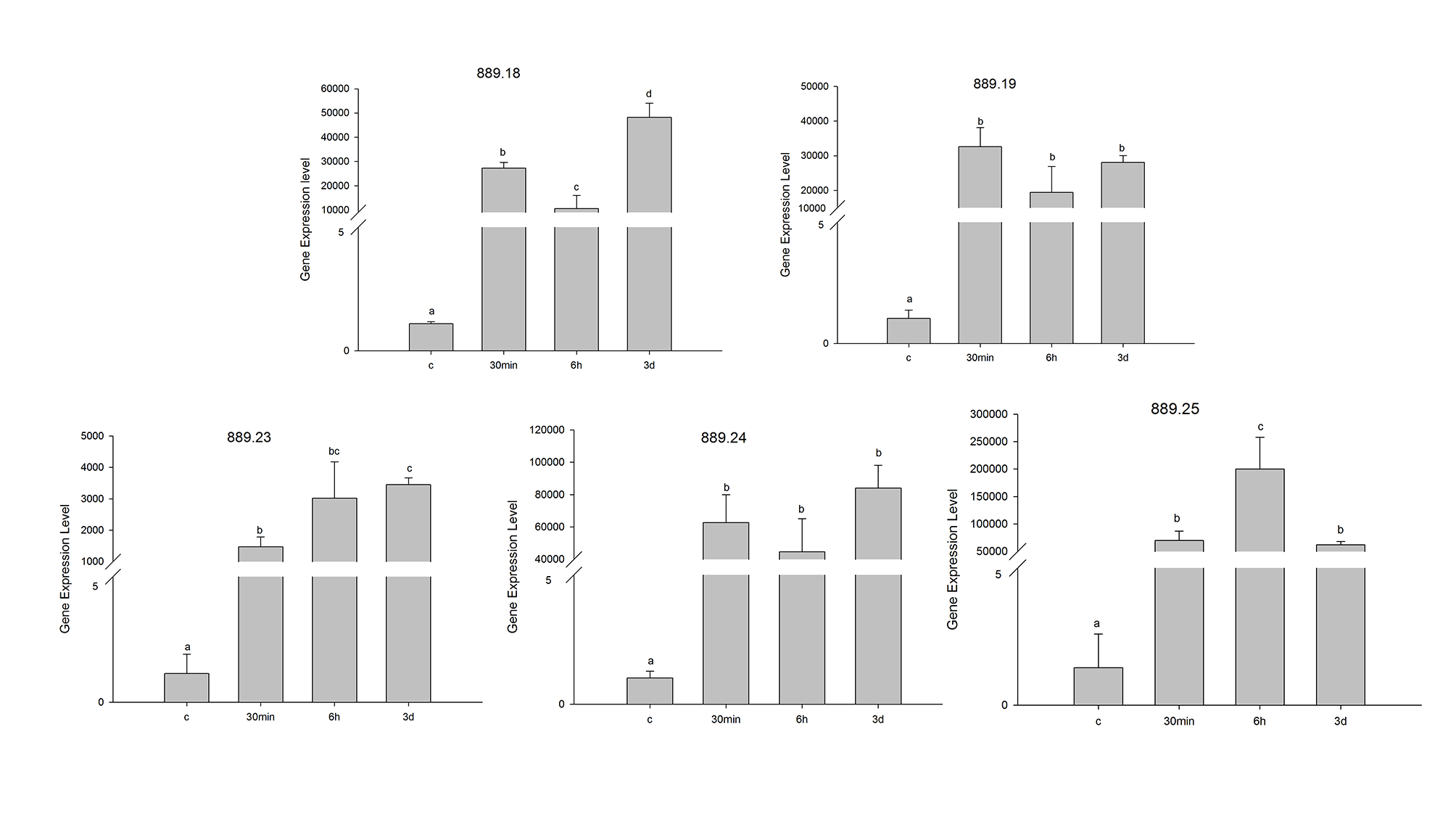

Supplement: S14 Fig — The data are expressed as mean ± SD after normalization. (TIF) [file pbio.2003790.s017.tif]

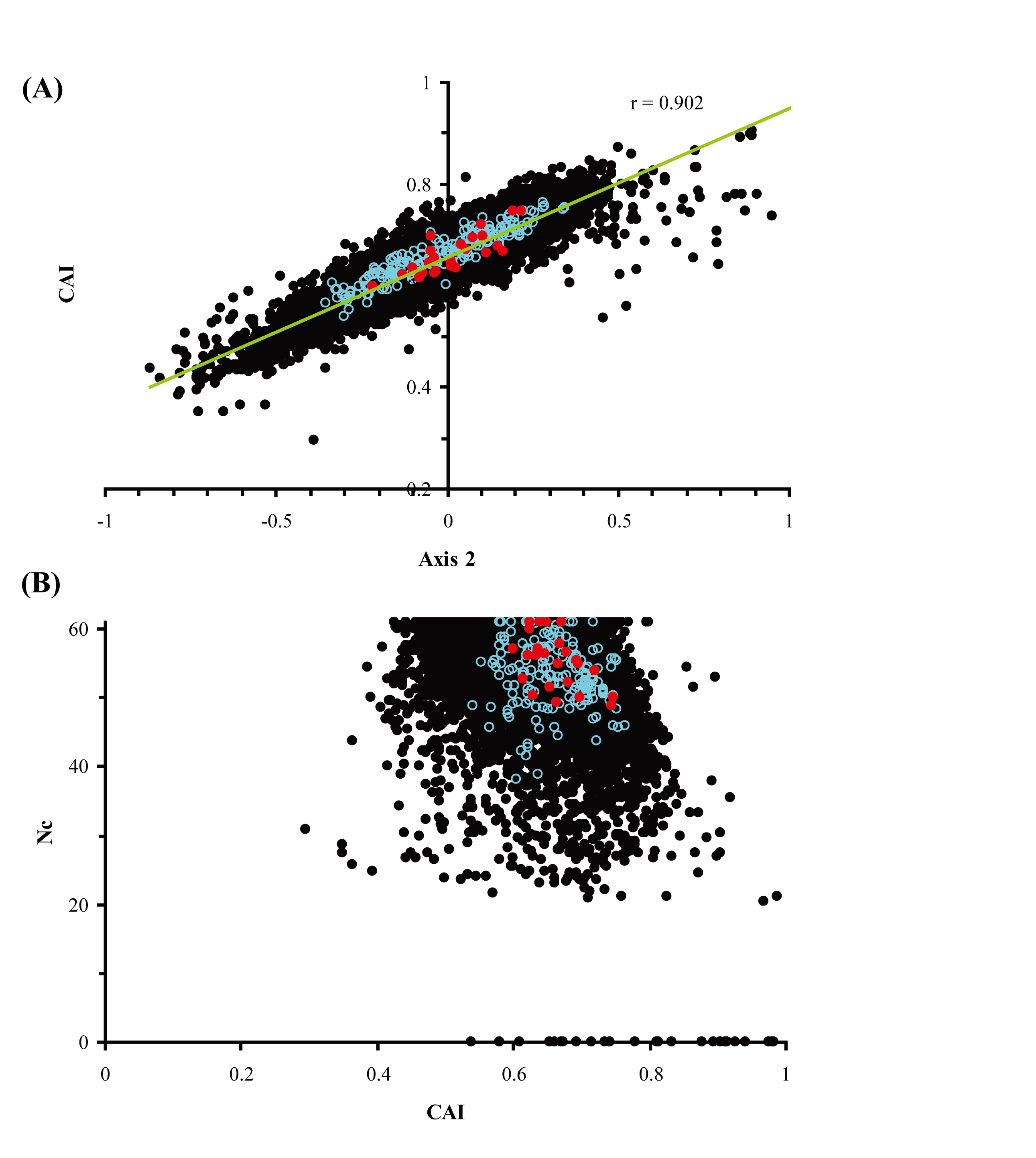

Supplement: S16 Fig — (A) Plot of the second important axis (Axis2) after correspondence analysis against the codon adaptive index (CAI). A strong positive correlation between Axis2 and CAI indicates that gene expression level is one of the major factors affecting codon usage. (B) Plot of CAI against the effective number of codons (Nc). Blue circles stand for the genes encoding ribosomal proteins, which are commonly considered as highly expressed proteins. Red circles stand for 11 prostatic secretory protein of 94 amino acids (PSP94)-like genes. Like ribosomal proteins, PSP94-like genes showed high CAI and Nc values, suggesting that PSP94-like genes are also highly expressed genes. (TIF) [file pbio.2003790.s019.tif]

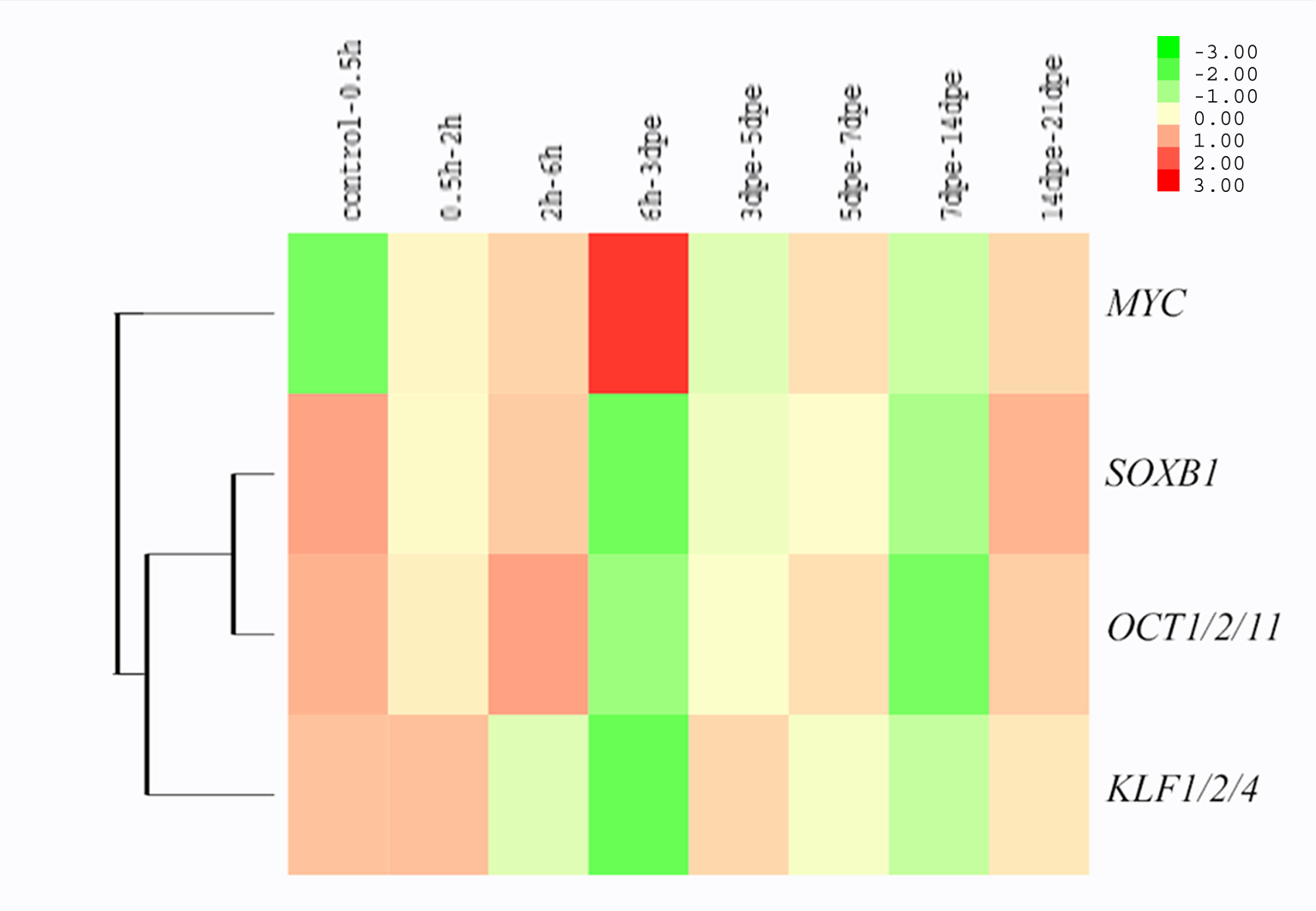

Supplement: S17 Fig — The orthologs of the 4 key pluripotency factors in A. japonicus were SoxB1 (AJAP16673), Myc (AJAP11986), Oct1/2/11 (AJAP10188), and Klf1/2/4 (AJAP08993). Color illustrates the fold changes of gene expression level between adjacent regeneration stages. Red represents up-regulation, and green represents down-regulation. (TIF) [file pbio.2003790.s020.tif]
